# Supplementary material for: Co-infections of Schistosoma mansoni and Helicobacter pylori in school-aged populations and implication for management and control practices in Niger State, Nigeria
Source: PLoS One. 2025 Dec 8;20(12):e0337423. doi: 10.1371/journal.pone.0337423 (PMC12685161; doi:10.1371/journal.pone.0337423)
Supplement: S2 File — (DOCX) [file pone.0337423.s002.docx]

STROBE Statement—Checklist of items that should be included in reports of ***cross-sectional studies***

|  | Item No | Recommendation | Page No |
| --- | --- | --- | --- |
| **Title and abstract** | 1 | (*a*) Indicate the study’s design with a commonly used term in the title or the abstract | Page 2, line 26 |
|  |  | (*b*) Provide in the abstract an informative and balanced summary of what was done and what was found | Page 2, line 26-43 |
| Introduction | | | |
| Background/rationale | 2 | Explain the scientific background and rationale for the investigation being reported | Page 4, line 68-91 |
| Objectives | 3 | State specific objectives, including any prespecified hypotheses | Page 4, line 89-91 |
| Methods | | | |
| Study design | 4 | Present key elements of study design early in the paper | Page 5, line 108-116 |
| Setting | 5 | Describe the setting, locations, and relevant dates, including periods of recruitment, exposure, follow-up, and data collection | Page 5, line 108-116 |
| Participants | 6 | (*a*) Give the eligibility criteria, and the sources and methods of selection of participants | Page 6, line 119-129 |
| Variables | 7 | Clearly define all outcomes, exposures, predictors, potential confounders, and effect modifiers. Give diagnostic criteria, if applicable | Page 6, lines 130-135 |
| Data sources/ measurement | 8* | For each variable of interest, give sources of data and details of methods of assessment (measurement). Describe comparability of assessment methods if there is more than one group | *Page 6-8, lines 130-165* |
| Bias | 9 | Describe any efforts to address potential sources of bias | Page 7, line 142-144 |
| Study size | 10 | Explain how the study size was arrived at | Page 6, 119-123 |
| Quantitative variables | 11 | Explain how quantitative variables were handled in the analyses. If applicable, describe which groupings were chosen and why | Page 8, lines 167-175 |
| Statistical methods | 12 | (*a*) Describe all statistical methods, including those used to control for confounding | Page 8, lines 167-175 |
|  |  | (*b*) Describe any methods used to examine subgroups and interactions | Page 8, lines 167-175 |
|  |  | (*c*) Explain how missing data were addressed | Page 10 Page 8, lines 167-175 |
|  |  | (*d*) If applicable, describe analytical methods taking account of sampling strategy | NA |
|  |  | (*e*) Describe any sensitivity analyses | NA |
| Results | | | |
| Participants | 13* | (a) Report numbers of individuals at each stage of study—eg numbers potentially eligible, examined for eligibility, confirmed eligible, included in the study, completing follow-up, and analysed | Page 8-9, lines 178-191 |
|  |  | (b) Give reasons for non-participation at each stage | NA |
|  |  | (c) Consider use of a flow diagram | NA |
| Descriptive data | 14* | (a) Give characteristics of study participants (eg demographic, clinical, social) and information on exposures and potential confounders | P Page 8-9, lines 178-191 |
|  |  | (b) Indicate number of participants with missing data for each variable of interest | NA |
| Outcome data | 15* | Report numbers of outcome events or summary measures | Page 9-11, Lines 192-216 |
| Main results | 16 | (*a*) Give unadjusted estimates and, if applicable, confounder-adjusted estimates and their precision (eg, 95% confidence interval). Make clear which confounders were adjusted for and why they were included | Page 10-11, lines 207-220 |
|  |  | (*b*) Report category boundaries when continuous variables were categorized | NA |
|  |  | (*c*) If relevant, consider translating estimates of relative risk into absolute risk for a meaningful time period | NA |
| Other analyses | 17 | Report other analyses done—eg analyses of subgroups and interactions, and sensitivity analyses | Page 8-11, lines 178-220 |
| Discussion | | | |
| Key results | 18 | Summarise key results with reference to study objectives | Page 12-14, lines 222-269 |
| Limitations | 19 | Discuss limitations of the study, taking into account sources of potential bias or imprecision. Discuss both direction and magnitude of any potential bias | Page 12-14, lines 222-269 |
| Interpretation | 20 | Give a cautious overall interpretation of results considering objectives, limitations, multiplicity of analyses, results from similar studies, and other relevant evidence | Page 12-14, lines 222-269 |
| Generalisability | 21 | Discuss the generalisability (external validity) of the study results | Page 12-14, lines 222-269 |
| Other information | | | |
| Funding | 22 | Give the source of funding and the role of the funders for the present study and, if applicable, for the original study on which the present article is based | Page 15, Line 290-292 |

*Give information separately for exposed and unexposed groups.

**Note:** An Explanation and Elaboration article discusses each checklist item and gives methodological background and published examples of transparent reporting. The STROBE checklist is best used in conjunction with this article (freely available on the Web sites of PLoS Medicine at http://www.plosmedicine.org/, Annals of Internal Medicine at http://www.annals.org/, and Epidemiology at http://www.epidem.com/). Information on the STROBE Initiative is available at www.strobe-statement.org.
