# Supplementary material for: Co-infections of Schistosoma mansoni and Helicobacter pylori in school-aged populations and implication for management and control practices in Niger State, Nigeria
Source: PLoS One. 2025 Dec 8;20(12):e0337423. doi: 10.1371/journal.pone.0337423 (PMC12685161; doi:10.1371/journal.pone.0337423)
Supplement: Attachment — Submitted filename: PONE-D-25-40863 AUGUST 2025.pdf [file pone.0337423.s004.pdf]

PLOS ONE

Co-infections of Schistosoma mansoni and Helicobacter pylori in School-Aged Populations and implication for management and control practices

--Manuscript Draft--

**Manuscript Number:**	PONE-D-25-40863
**Article Type:**	Research Article
**Full Title:**	Co-infections of Schistosoma mansoni and Helicobacter pylori in School-Aged Populations and implication for management and control practices
**Short Title:**	Schistosoma mansoni and Helicobacter pylori in School-Aged Populations
**Corresponding Author:**	Hammed Oladeji Mogaji, Ph.D<br>Marian University<br>UNITED STATES OF AMERICA
**Keywords:**	Schistosoma mansoni; H. pylori; Association; School-aged children; Nigeria
**Abstract:**	Schistosoma mansoni is a chronic human intestinal parasite that is targeted for elimination through mass drug administration of praziquantel. Helicobacter pylori, a pathogenic bacterium that also colonizes the gastrointestinal tract, but the understanding of how co-infections impact disease pathology is poorly understood. Hence, we conducted a cross-sectional study to investigate the co-infections of both pathogens among school-aged population sampled across five study communities in Niger area of Nigeria. Fresh stool samples were obtained from 299 school-aged children between ages 6-17 years and analyzed using real-time and conventional polymerase chain reaction (PCR) for the direct detection of S. mansoni and H. pylori respectively. An overall prevalence of 19.7% (4.5% - 24.1%) was recorded for H. pylori, and 34.4% (0% - 42.1%) for S. mansoni, while co-infection of both was 7.0% (0% - 8.1%). Infections were significantly different across the study communities for S. mansoni ($p<0.05$) when compared to H. pylori ($p > 0.38$). There were, however, no significant association between infection and gender ($p > 0.05$), with the following odds of infection for S. mansoni (OR=0.69, 95% CI: 0.42, 1.12), H. pylori (OR=1.33, 95% CI: 0.75, 2.36), and combination of both infections (OR=1.31, 95% CI: 0.83, 2.09). By age category, children below 14years were twice likely to be exposed to the combination of both infections; age 12-14 years (OR=1.9, 95% CI: 1.06, 3.44), and age 9-11 years (OR=1.96, 95% CI: 1.11, 3.48). But they were also less likely to be exposed to S. mansoni; age 12-14 years (OR=0.52, 95% CI: 0.27, 0.97), and age 9-11 years (OR=0.43, 95% CI: 0.23, 0.79). While the co-infection rate is low, the findings on S. mansoni are particularly concerning, which underscore the need for integrated interventions, including chemotherapy and improved water, sanitation, and hygiene (WASH) services.
**Order of Authors:**	Muinah Fowora
	Babatunde Adewale
	Hammed Oladeji Mogaji, Ph.D
	Omolola Omolopo
	Balogun Daniel
	Abdulrahman Aladejana
	De'Broski R. Herbert
**Additional Information:**	
**Question**	**Response**
**Financial Disclosure**<br><br>Enter a financial disclosure statement that describes the sources of funding for the	Yes

Powered by Editorial Manager® and ProduXion Manager® from Aries Systems Corporation

work included in this submission. Review the submission guidelines for detailed requirements. View published research articles from *PLOS ONE* for specific examples.<br><br>This statement is required for submission and **will appear in the published article** if the submission is accepted. Please make sure it is accurate.<br><br>**Funded studies**<br>Enter a statement with the following details:<br>• Initials of the authors who received each award<br>• Grant numbers awarded to each author<br>• The full name of each funder<br>• URL of each funder website<br>• Did the sponsors or funders play any role in the study design, data collection and analysis, decision to publish, or preparation of the manuscript?<br><br>Did you receive funding for this work?	
Please add funding details.<br>as follow-up to "**Financial Disclosure**<br><br>Enter a financial disclosure statement that describes the sources of funding for the work included in this submission. Review the submission guidelines for detailed requirements. View published research articles from *PLOS ONE* for specific examples.<br><br>This statement is required for submission and **will appear in the published article** if the submission is accepted. Please make sure it is accurate.	This work received partial support from the Nigerian Institute of Medical Research Grant for studies on Neglected Tropical Diseases [Grant number 0000134].

**Funded studies**<br>Enter a statement with the following details:<br>• Initials of the authors who received each award<br>• Grant numbers awarded to each author<br>• The full name of each funder<br>• URL of each funder website<br>• Did the sponsors or funders play any role in the study design, data collection and analysis, decision to publish, or preparation of the manuscript?<br><br>Did you receive funding for this work?"	
Please select the country of your main research funder (please select carefully as in some cases this is used in fee calculation).<br>as follow-up to "**Financial Disclosure**<br><br>Enter a financial disclosure statement that describes the sources of funding for the work included in this submission. Review the submission guidelines for detailed requirements. View published research articles from *PLOS ONE* for specific examples.<br><br>This statement is required for submission and **will appear in the published article** if the submission is accepted. Please make sure it is accurate.<br><br>**Funded studies**<br>Enter a statement with the following details:<br>• Initials of the authors who received each award<br>• Grant numbers awarded to each author<br>• The full name of each funder<br>• URL of each funder website<br>• Did the sponsors or funders play any role in the study design, data collection and analysis, decision to publish, or preparation of the manuscript?<br><br>Did you receive funding for this work?"	NIGERIA - NG
**Competing Interests**	The authors have declared that no competing interests exist.

Use the instructions below to enter a competing interest statement for this submission. On behalf of all authors, disclose any competing interests that could be perceived to bias this work—acknowledging all financial support and any other relevant financial or non-financial competing interests.<br><br>This statement is required for submission and **will appear in the published article** if the submission is accepted. Please make sure it is accurate and that any funding sources listed in your Funding Information later in the submission form are also declared in your Financial Disclosure statement.<br><br>View published research articles from *PLOS ONE* for specific examples.<br><br>**NO authors have competing interests**<br><br>Enter: *The authors have declared that no competing interests exist.*<br><br>**Authors with competing interests**<br><br>Enter competing interest details beginning with this statement:<br><br>*I have read the journal's policy and the authors of this manuscript have the following competing interests: [insert competing interests here]*<br><br>* typeset	
**Ethics Statement**<br><br>Enter an ethics statement for this submission. This statement is required if the study involved:<br><br>• Human participants<br>• Human specimens or tissue<br>• Vertebrate animals or cephalopods<br>• Vertebrate embryos or tissues	The study received approval from the Nigerian Institute of Medical Research ethical review board (IRB/18/042). Informed consent was obtained from parents/guardian and children. Children's assent was obtained verbally and documented through an assent form in the presence of the parent or legal guardian. Unique identifiers and a password-protected database were also used to ensure anonymity and confidentiality through the study procedures. The study procedures were implemented following the ethical standards of the Helsinki Declaration (1964, amended most recently in 2008) of the World Medical Association.

• Field research<br><br>Write "N/A" if the submission does not require an ethics statement.<br><br>General guidance is provided below. Consult the submission guidelines for detailed instructions. **Make sure that all information entered here is included in the Methods section of the manuscript.**	

Powered by Editorial Manager® and ProduXion Manager® from Aries Systems Corporation

**Format for specific study types**<br><br>**Human Subject Research (involving human participants and/or tissue)**<br>• Give the name of the institutional review board or ethics committee that approved the study<br>• Include the approval number and/or a statement indicating approval of this research<br>• Indicate the form of consent obtained (written/oral) or the reason that consent was not obtained (e.g. the data were analyzed anonymously)<br><br>**Animal Research (involving vertebrate animals, embryos or tissues)**<br>• Provide the name of the Institutional Animal Care and Use Committee (IACUC) or other relevant ethics board that reviewed the study protocol, and indicate whether they approved this research or granted a formal waiver of ethical approval<br>• Include an approval number if one was obtained<br>• If the study involved *non-human primates*, add *additional details* about animal welfare and steps taken to ameliorate suffering<br>• If anesthesia, euthanasia, or any kind of animal sacrifice is part of the study, include briefly which substances and/or methods were applied<br><br>**Field Research**<br><br>Include the following details if this study involves the collection of plant, animal, or other materials from a natural setting:<br>• Field permit number<br>• Name of the institution or relevant body that granted permission	
**Data Availability**<br><br>Authors are required to make all data underlying the findings described fully available, without restriction, and from the time of publication. PLOS allows rare exceptions to address legal and ethical concerns. See the PLOS Data Policy and FAQ for detailed information.	Yes - all data are fully available without restriction

A Data Availability Statement describing where the data can be found is required at submission. Your answers to this question constitute the Data Availability Statement and **will be published in the article**, if accepted.<br><br>**Important:** Stating 'data available on request from the author' is not sufficient. If your data are only available upon request, select 'No' for the first question and explain your exceptional situation in the text box.<br><br>Do the authors confirm that all data underlying the findings described in their manuscript are fully available without restriction?	
**Describe where the data may be found in full sentences. If you are copying our sample text, replace any instances of XXX with the appropriate details.**<br><br>• If the data are **held or will be held in a public repository**, include URLs, accession numbers or DOIs. If this information will only be available after acceptance, indicate this by ticking the box below. For example: *All XXX files are available from the XXX database (accession number(s) XXX, XXX.*).<br>• If the data are all contained **within the manuscript and/or Supporting Information files**, enter the following: *All relevant data are within the manuscript and its Supporting Information files.*<br>• If neither of these applies but you are able to provide **details of access elsewhere**, with or without limitations, please do so. For example:<br><br>*Data cannot be shared publicly because of [XXX]. Data are available from the XXX Institutional Data Access / Ethics Committee (contact via XXX) for researchers who meet the criteria for access to confidential data.*<br><br>*The data underlying the results presented in the study are available from (include the name of the third party*	The datasets used and/or analyzed are available as supplementary files

and contact information or URL).<br>• This text is appropriate if the data are owned by a third party and authors do not have permission to share the data.<br><br>* typeset	
Additional data availability information:	

Powered by Editorial Manager® and ProduXion Manager® from Aries Systems Corporation

Manuscript

Click here to access/download;Manuscript;Helico Schisto Manuscript final_MF Revised.docx

Co-infections of *Schistosoma mansoni* and *Helicobacter pylori* in School-Aged Populations and implication for management and control practices

Muinah Fowora[1], Babatunde Adewale[2], Hammed Mogaji [2,3,4]*, Omolola Omolopo[1], Balogun Daniel[2], Abdulrahman Aladejana[5], and De'Broski R. Herbert[6]

[1]*Molecular Biology and Biotechnology Department, Nigerian Institute of Medical Research Yaba, Lagos Nigeria*.

[2]Public Health and Epidemiology Department, Nigerian Institute of Medical Research, Yaba, Lagos Nigeria.

[3]Public Health Unit, Department of Applied and Behavioral Sciences, College of Arts and Sciences, Marian University, United States of America

[4]Department of Research and Development, Mission To Safe The Helpless, Lagos State, Nigeria

[5]Department of Animal and Environmental Biology, Federal University Oye-Ekiti, Ekiti State, Nigeria

[6]Department of Microbiology and Immunology, Tulane University School of Medicine, New Orleans, LA, United States of America

Corresponding author: *mogajihammed@gmail.com

Keywords: *Schistosoma mansoni*, *H. pylori*, Association, School-aged children, Nigeria

Abstract

Schistosoma mansoni is a chronic human intestinal parasite that is targeted for elimination through mass drug administration of praziquantel. *Helicobacter pylori*, a pathogenic bacterium that also colonizes the gastrointestinal tract, but the understanding of how co-infections impact disease pathology is poorly understood. Hence, we conducted a cross-sectional study to investigate the co-infections of both pathogens among school-aged population sampled across five study communities in Niger area of Nigeria. Fresh stool samples were obtained from 299 school-aged children between ages 6-17 years and analyzed using real-time and conventional polymerase chain reaction (PCR) for the direct detection of *S. mansoni* and *H. pylori* respectively. An overall prevalence of 19.7% (4.5% - 24.1%) was recorded for *H. pylori*, and 34.4% (0% - 42.1%) for *S. mansoni,* while co-infection of both was 7.0% (0% - 8.1%). Infections were significantly different across the study communities for *S. mansoni* ($p<0.05$) when compared to *H. pylori* ($p > 0.38$). There were, however, no significant association between infection and gender ($p > 0.05$), with the following odds of infection for *S. mansoni* (OR=0.69, 95% CI: 0.42, 1.12), *H. pylori* (OR=1.33, 95% CI: 0.75, 2.36), and combination of both infections (OR=1.31, 95% CI: 0.83, 2.09). By age category, children below 14years were twice likely to be exposed to the combination of both infections; age 12-14 years (OR=1.9, 95% CI: 1.06, 3.44), and age 9-11 years (OR=1.96, 95% CI: 1.11, 3.48). But they were also less likely to be exposed to *S. mansoni*; age 12-14 years (OR=0.52, 95% CI: 0.27, 0.97), and age 9-11 years (OR=0.43, 95% CI: 0.23, 0.79). While the co-infection rate is low, the findings on *S. mansoni* are particularly concerning, which underscore the need for integrated interventions, including chemotherapy and improved water, sanitation, and hygiene (WASH) services.

Keywords: *Helicobacter pylori, S. mansoni*, Chemotherapy, Nigerian, Elimination

Introduction

Schistosomiasis is one of the most prevalent neglected tropical diseases in the world with more than 200 million persons affected worldwide across 78 countries, mostly in areas where access to water, sanitation and hygiene facilities are lacking [1-4]. The disease is caused by water-borne trematode parasites of the genus Schistosoma, with two major species of *Schistosoma*; the first is the *S. haematobium* which inhabits the vesicular and pelvic venous plexus of the bladder and causes urogenital schistosomiasis and the second is *S. mansoni* which is more often in the inferior mesenteric veins draining the large intestine and causes intestinal schistosomiasis [2-4]. Schistosomiasis is a focal disease, with urogenital schistosomiasis been more prevalent, and perhaps more studied in endemic settings compared to, intestinal schistosomiasis [5]. Intestinal schistosomiasis has severe acute complications associated with the intestine - abdominal pain, diarrhea, blood in the stool, and in more severe cases, could lead to chronic enlargement of the liver and spleen, a condition known as hepatosplenomegaly [5-8]. The pathologies are worsened among children because of the developing immunity, with already established evidence on anemia, stunting, protein-energy malnutrition, school absenteeism and reduced cognition [9-10].

People infected with *S. mansoni* may be infected with *Helicobacter pylori* (*H. pylori*), a Gram-negative bacterium that also colonizes the gastrointestinal tract [11,12]. *H. pylori* is the most common human bacterium, and causative agent of gastritis, peptic ulcer disease, mucosa associated lymphoid tissue lymphoma and gastric cancer (GC) [13]. This bacterium infects 50% of the world's population, with about 80% of African population affected [13]. The risk factors for *H. pylori* acquisition have been reported to be mainly due to overcrowding, to have infected

siblings or parent and to unsafe water sources [13]. Both *H. pylori* and *S mansoni* share similar risk been that they both infect the gastrointestinal tract, could be acquired earlier in childhood, can remain asymptomatic for a long period [7,8,13]. In regions where co-infections occur, there are overlapping clinical manifestations, with extra-gastroduodenal manifestations such as chronic liver diseases and iron-deficiency anemia [14-15]. There are also speculations of altered disease pathology, either as synergistic or antagonistic interactions, which may influence treatment and clinical outcomes, and susceptibility to other diseases [16].

Efforts targeted at controlling schistosomiasis have been ongoing for over a decade in Africa, and other endemic settings with routine diagnosis and administration of large scale praziquantel medications [1-3], there are currently no guidelines targeted at diagnosing or controlling *H. pylori* infections within routine primary health care programs [13]. In Nigeria, the most populous country in Africa, several studies have reported the co-infection of *H. pylori* with intestinal parasites and soil transmitted helminthes and its associated burden [17, 18], but studies on co-infections of *H. pylori* and *S. mansoni* are scarce. This evidence is necessary to support improvement of management strategies. This study therefore reports the prevalence and associated risk factors of *H. pylori* and *S. mansoni* co-infection among school-aged population across five study communities in Niger State of Nigeria.

Materials and Methods

Ethical Statement and Considerations

The study received approval from the Nigerian Institute of Medical Research ethical review board (IRB/18/042). Informed consent was obtained from parents/guardian and children. Children's assent was obtained verbally and documented through an assent form in the presence of the parent or legal guardian. Unique identifiers and a password-protected database were also used to ensure anonymity and confidentiality through the study procedures. The study procedures were implemented following the ethical standards of the Helsinki Declaration (1964, amended most recently in 2008) of the World Medical Association.

Study area, design and selection of sampling sites

This study was conducted between 1st and 30th of April 2019, as part of a larger project, with the area and selection procedure previously described [19]. Briefly, five communities (Monai, Tamanai, Koro, Musarwa, and Yuna) perceived to be at risk of water-borne infections were purposively selected based on proximity to Kainji Dam in Borgu LGA of Niger state. Majority of the populations in these communities engage in farming, fishing, and trading, and there is limited access to improved sources of water and latrine facilities. We employed a cross-sectional approach to administer pretested questionnaires and obtain fresh stool sample collection from school-aged children between ages 6-16 years.

Sample Size Determination and Selection of Study Participants

Participants recruited in this study were subset of those recruited in the larger study [19]. Four hundred and ten pupils in close proximity communities to Kainji Dam were enlisted following their informed consent. The sample size was calculated using the sample size estimate from a National survey with a 95% confidence interval[11]. Information on the age, sex and water contact activities of children was collected using structured questionnaires. Children within the age range of 5–16 years, who had not received any anti-helminth treatment within the last 6 months, were recruited from the communities for the study. Summarily, 410 children were enrolled in the larger study, only 22/133 from Monai, 79/79 from Tamanai, 124/124 from Koro, 36/36 from Musawa, and 38/38 from Yuna, participated in this study.

Questionnaire Administration

Using semi-structured questionnaires each pupil was allotted a unique code of six digits representing community, class and serial numbers while demographic data includes the name, age, sex, and parents' occupation were recorded using a simple field and assigned participants ID on both consent/assent form, field form and stool specimen bottle. The field form was designed in the English language but administered in Hausa language.

Collection of Stool Samples

Participants were provided with one sterile specimen bottle pre-labelled with their unique identification number, an applicator stick, a plain sheet of paper, and tissue paper to clean their

anus. Participants were instructed to defecate on the plain sheet of paper and use the applicator stick to transfer a fresh portion into the specimen bottle. Specimen bottles were retrieved within 1 h of distribution, but collection was between 10 h and 14 h. All collected stool samples were sorted and transported in iceboxes for processing within 2 hours of collection to the NIMR outstation laboratory located in Kainji.

Laboratory processing of stool samples

The endemicity was classified based on the aggregated prevalence in each community. For H. pylori identification, stored stool sample in ice-boxes were transported to the Molecular Biology and Biotechnology Department of the Nigerian Institute of Medical Research. Genomic DNA was extracted and purified from the stool samples using NIMR Biotech stool DNA extraction kit in accordance with the manufacturer's instructions. An initial Polymerase Chain Reaction (PCR) was carried out using *Helicobacter* species specific primers as previously described [20]. All samples positive for the presence of *Helicobacter* species were further analyzed with a second PCR targeting the 16S rRNA gene of *H pylori*. The second PCR reaction was carried out using the primer set Hpy-F (5′-CGC ACC TGC TGG AAC ATT AC-3′) and Hpy-R (5′-CGT TAG CTG CAT TAC TGG AGA-3′) as previously described [21]. All PCR fragments were separated on a 2% agarose gel and visualized after ethidium bromide staining. A 138-bp fragment confirmed the presence of *H. pylori* in the stool sample.

A real time-PCR was used to detect cell-free *S. mansoni* in the DNA samples. The reaction was carried out using the set of primers and probes that amplifies a 121 bp tandem repeat sequence of *S. mansoni* strain SM 1-7 (GenBank accession number: M61098) as previously described [22, 23].

The primer and probe sequence were Sm FW 5′-CCG ACC AAC CGT TCT ATG A-3′; Sm RV 5′-CAC GC TCT CG C AAA TAA TCT AAA-3′; Sm probe 5′-[FAM] TCG TTG TAT CTC CGA AAC CAC TGG ACG [(BHQ1])-3′ all synthesized by Biomers GMBH, Germany.

Data Management and Analysis

Data obtained from this study were imported and analysed in R. software version 4.3.2 (S1 File). Descriptive statistics, including frequencies and percentages, were used to describe the prevalence and co-infection patterns, and tested association across communities using chi-square statistics. Significant associations were established when $p < 0.05$. Furthermore, we performed three univariate logistic regression model each between single (*H. pylori, S mansoni*), co-infection (*H pylori* and *S. mansoni*), and demographic factor (sex and age). Regression estimates were reported in the form of odds ratios (OR) and 95% confidence intervals (CI). The significance level was established as $p < 0.05$.

Results

Demographic Characteristics of Study Participants

Table 1 presents the age and sex distribution of the 299 participants recruited for the study from five communities: Monai (n=22, 7.4%), Tamani (n=79, 26.4%), Koro (n=124, 41.5%), Musawa (n=36, 12.0%), and Yuna (n=38, 12.7%). Male participants (n=174, 58.2%) were more than females (n=125, 41.8%). The age group distribution showed that the majority of participants were aged 6-8 years (n=96, 32.1%), followed by 9-11 years (n=102, 34.1%), 12-14 years (n=90, 30.1%), and 15-17 years (n=11, 3.7%). Significant differences were observed in the age and sex distribution

across the communities ($p < 0.001$). Monai presented the highest proportion of participants aged 6-8 years (21, 95.5%), while Yuna had the highest proportion in the 12-14 years category (25, 65.8%). Males were significantly more represented in Yuna (34, 89.5%) compared to other communities ($p < 0.001$).

Table 1: Sex and age characteristics of the study population

		Communities					
	**N = 299**	**Monai (N = 22)**	**Tamani (N = 79)**	**Koro (N = 124)**	**Musawa (N = 36)**	**Yuna (N = 38)**	**p-value**
**age, n (%)**							<0.001
6-8yrs	96 (32.1%)	21 (95.5%)	5 (6.3%)	41 (33.1%)	22 (61.1%)	7 (18.4%)	
9-11yrs	102 (34.1%)	1 (4.5%)	43 (54.4%)	38 (30.6%)	14 (38.9%)	6 (15.8%)	
12-14yrs	90 (30.1%)	0 (0.0%)	26 (32.9%)	39 (31.5%)	0 (0.0%)	25 (65.8%)	
15-17yrs	11 (3.7%)	0 (0.0%)	5 (6.3%)	6 (4.8%)	0 (0.0%)	0 (0.0%)	
**sex, n (%)**							<0.001
Female	125 (41.8%)	8 (36.4%)	41 (51.9%)	56 (45.2%)	16 (44.4%)	4 (10.5%)	
Male	174 (58.2%)	14 (63.6%)	38 (48.1%)	68 (54.8%)	20 (55.6%)	34 (89.5%)	

Prevalence of *Helicobacter pylori* and *Schistosoma mansoni* Infections

Table 2 summarizes the prevalence of *H. pylori and S. mansoni* infections among the participants. An overall prevalence of 19.7% was recorded for *Helicobacter pylori* across the communities. Tamani (24.1%) had the highest prevalence, while Monai (4.5%) had the lowest prevalence. However, there were no statistically significant differences in *H. pylori* prevalence across the communities ($p = 0.38$). In contrast, *S. mansoni* had a significantly higher overall prevalence of 34.4%. Yuna (42.1%), closely followed by Koro (41.1%) had the highest prevalence across

communities, while Monai reported no cases (0.0%). The differences in *S. mansoni* prevalence across communities were statistically significant ($p < 0.001$). Participants were further categorized based on their infection status: no infection (52.8%), mono-infection (40.1%), or co-infection (7.0%). Co-infection rates were highest in Koro (8.1%), followed by Yuna (7.9%), while Monai had (0%) coinfection rate. These results were statistically different among the communities (p = 0.004).

Table 2: Prevalence of *H. pylori and S. mansoni* infection among the study population

	N = 299	Monai (N = 22)	Tamani (N = 79)	Koro (N = 124)	Musawa (N = 36)	Yuna (N = 38)	p-value
H. pylori							0.38
Positive	59 (19.7%)	1 (4.5%)	19 (24.1%)	24 (19.4%)	7 (19.4%)	8 (21.1%)	
Negative	240 (80.3%)	21 (95.5%)	60 (75.9%)	100 (80.6%)	29 (80.6%)	30 (78.9%)	
S. mansoni							<0.001
Positive	103 (34.4%)	0 (0.0%)	29 (36.7%)	51 (41.1%)	7 (19.4%)	16 (42.1%)	
Negative	196 (65.6%)	22 (100.0%)	50 (63.3%)	73 (58.9%)	29 (80.6%)	22 (57.9%)	
**Category**							0.004
None	158 (52.8%)	21 (95.5%)	37 (46.8%)	59 (47.6%)	24 (66.7%)	17 (44.7%)	
Mono	120 (40.1%)	1 (4.5%)	36 (45.6%)	55 (44.4%)	10 (27.8%)	18 (47.4%)	
Double	21 (7.0%)	0 (0.0%)	6 (7.6%)	10 (8.1%)	2 (5.6%)	3 (7.9%)	

Association Between Infections and Demographic Factors

Table 3 shows the association between infections and demographic variables. Compared to females, males had non-significant associations with *H. pylori* (OR: 1.33, 95% CI: 0.75–2.36, p =0.33), *S. mansoni* (OR: 0.69, 95% CI: 0.42–1.12, p =0.14) and co-infection (OR: 1.31, 95% CI: 0.83–2.09, p =0.25). By age, participants aged 9-11 and 12-14years had significant lower odds of infection with *S. mansoni* (OR: 0.43, 95% CI: 0.23–0.79, p = 0.01) and (OR: 0.52, 95% CI: 0.27–0.97, p = 0.04), respectively when compared to those aged 6-8 years. However, the odds of co-

infection with *H. pylori and S. mansoni* were significantly higher among participants aged 9-11 years (OR: 1.96, 95% CI: 1.11–3.48, p = 0.02) and 12-14 years (OR: 1.90, 95% CI: 1.06–3.44, p = 0.03) compared to those aged 6-8 years.

Table 3: Association between infections and participants demographic

	**Pathogens**					
	H. pylori		***S. mansoni***		**Co-infection**	
	OR (95% CI)	p-value	OR (95% CI)	p-value	OR (95% CI)	p-value
**sex, n (%)**						
Female	1		1		1	
Male	1.33 (0.75, 2.36)	0.33	0.69 (0.42, 1.12)	0.14	1.31 (0.83, 2.09)	0.25
**age, n (%)**						
6-8yrs	1		1		1	
9-11yrs	0.77(0.37, 1.58)	0.48	0.43(0.23, 0.79)	0.01	1.96 (1.11, 3.48)	0.02
12-14yrs	0.75 (0.35, 1.56)	0.44	0.52 (0.27, 0.97)	0.04	1.9 (1.06, 3.44)	0.03
15-17yrs	0.53 (0.14, 2.63)	0.39	0.84 (0.22, 4.07)	0.81	1.45 (0.39, 5.36)	0.56

OR: Odds Ratios; CI: Confidence Interval

Discussion

In this study, we have reported the prevalence of co-infection with *H. pylori and S. mansoni* infections among school-aged children in five communities in Northern Nigeria. Our findings have provided some form of evidence that might be important to guide future directions for more robust research and programmatic actions. Foremost, the overall prevalence recorded for *H. pylori* was 19.7%, which is similar those reported in Owerri (20%) [24], but much lower than those reported in Imo State (72%) [25], Kano (70% and 38%) [26,27]. The northern part of Nigeria has been known to be heavily impacted [28,29], owing to increased risk associated with overcrowding and limited access to water and sanitation facilities [24,25]. This report however, deviates from this notion, and disparities could be attributed to differences in diagnostic tools, sampling sites and study participants, or in fact, the hygiene standards of the latter two factors.

We observed *H. pylori* prevalence was particularly low in Monai compared to other communities which could suggest a difference in community-level sanitation practices or health interventions, or maybe linked to age, as about 96% of those in Monai were below age 8years. However, we couldn't find any association between mono-infections with *H. pylori* based on age. We presume our limited sample-size and covariate frame might have impacted the identification of any meaningful association. However, our regression model (for combined infection) further emphasized increased risk of infection in older children compared to younger ones. Previous studies have reported in consonance that the risk of infections is increased among older participants [25,26,30,31]. A possible explanation for the higher rate of infection among older children is that exposures and reinfection are likely going to persist in areas where WASH services are limited and

socio-economic status are low, both condition which reflect the nature of our study location [30,31,32].

For *S. mansoni*, an overall prevalence of 34.4% (19.4% - 42.1%) was recorded, this is quite high considering previous findings in Nigeria — 2.93% in Sokoto [33], 8.93% in Kano [34], 3.6% in Ogun [35], and 9% in Osun [36]. This presents a source of concern for control programs with questions on persistent transmission considering environmental suitability and ineffective control program [37,38], or on a more worrisome note, the efficacy of praziquantel on *S. mansoni* [39]. Infections also shared similar pattern with *H. pylori* mono infection and with literature [35,38,40], as older children were more infected, and none of the younger children in Monai were infected. The odds of infection, even though lower in younger children were seen to progressively increase in magnitude as children age. These findings are logical, as infection with schistosomes is related to behavioral patterns, most especially water contact practices which are more common among older children due to increased liberty to engage in risky behaviors, such as swimming, bathing and fetching in river bodies infested with biomphalaria snail species [35,38,40].

Our observation of a 7.0% co-infection rate is less concerning than initially anticipated. However, the high prevalence of *S. mansoni* alone was surprising and raises significant public health concerns, underscoring the need for enhanced chemotherapy and improved water, sanitation, and hygiene (WASH) services. Reports of synergistic and antagonistic immunological interactions between *S. mansoni* and *H. pylori* [16,41] prompt further questions about whether these pathogens might exhibit antagonistic relationships at the ecological or behavioral levels, potentially

influencing transmission dynamics. This could explain the lower-than-expected co-infection prevalence observed in this study. While our findings are not robust enough to conclusively address this hypothesis, the question merits further investigation to better understand the interplay between these two pathogens in co-endemic settings.

Conclusion

Our study highlights an unexpectedly high prevalence of *S. mansoni*, moderate prevalence of H. *pylori*, and very low coinfection prevalence for both. Our findings on *S. mansoni* is particularly concerning, suggesting potential challenges in control programs, that needs investigation. While the co-infection rate was lower than expected, the findings still underscore the need for integrated interventions, including chemotherapy and improved water, sanitation, and hygiene (WASH) services, to address both infections effectively, especially among older children.

Supplementary Files

S1 File: Dataset

Declarations

Authors' contribution

MF, BA, and HOM conceptualized the study and prepared the protocol, DH approved the protocol. BA and HOM coordinated field surveys. BD, AA were involved in data collection. MF and OO carried out the molecular analysis. HOM performed statistical analysis, and MF and HOM prepared the first draft of the manuscript. All authors contributed to the development of the final

manuscript and approved its submission.

Funding

This work received partial support from the Nigerian Institute of Medical Research Grant for studies on Neglected Tropical Diseases [Grant number 0000134].

Conflict of Interest

The authors declare that the research was conducted in the absence of any commercial or financial relationships that could be construed as a potential conflict of interest.

Availability of data and materials

The datasets used and/or analyzed are available as supplementary files

Acknowledgements

We are grateful to the community leaders across the study communities, and all the health workers who facilitated the implementation process.

References

1. World Health Organization (WHO). Ending the neglect to attain the Sustainable Development Goals: a road map for neglected tropical diseases 2021–2030. Geneva: World Health Organization; 2020. Licence: CC BY-NC-SA 3.0 IGO.

2. World Health Organization (WHO). Schistosomiasis. 2021. https://www.who.int/news-room/fact-sheets/detail/schistosomiasis. Accessed 20 September 2021.

3. World Health Organization (WHO). Helminth control in school-age children: a guide for managers of control programmes. 2021. https://www.who.int/neglected_diseases/resources/9789241548267/en/. Accessed 20 September 2021.

4. Midzi N, Mduluza T, Chimbar MJ, Tshuma C, Charimar L, et al. Distribution of schistosomiasis and soil transmitted helminthiasis in Zimbabwe: towards a national plan of action for control and elimination. *PLoS Negl Trop Dis*. 2014;**8**, e3014. pmid:25121489

5. McManus, D.P., Dunne, D.W., Sacko, M. *et al.* Schistosomiasis. *Nat Rev Dis Primers* **4**, 13 (2018). https://doi.org/10.1038/s41572-018-0013-8

6. Centre for Disease Control (CDC). Schistosomiasis. Laboratory identification of parasites of public health concern. https://www.cdc.gov/dpdx/schistosomiasis/index.html. Accessed 20 September 2021.

7. Costain AH, MacDonald AS, Smits HH. Corrigendum: Schistosome Egg Migration: Mechanisms, Pathogenesis and Host Immune Responses. *Front. Immunol*. 2019;**10**: 749. pmid:31031753

8. Jose RL. Acute schistosomiasis mansoni: revisited and reconsidered. Mem Inst Oswaldo Cruz, Rio de Janeiro. 2010;**105**(4):422–435. pmid:20721485

9. Guerrant RL, Leite AM, Pinkerton R, Medeiros PH, Cavalcante PA et al. Biomarkers of Environmental Enteropathy, Inflammation, Stunting, and Impaired Growth in Children in Northeast Brazil. *PLoS ONE*. 2016;**11**(9):e0158772. pmid:27690129; PubMed Central PMCID: PMC5045163.

10. Osakunor DNM, Woolhouse MEJ, Mutapi F. Paediatric schistosomiasis: What we know and what we need to know. *PLoS Negl Trop Dis*. 2018;**12**(2): e0006144. pmid:29420537

11. Ibrahim A, Ali YBM, Abdel-Aziz A, El-Badry AA. Helicobacter pylori and enteric parasites co-infection among diarrheic and non-diarrheic Egyptian children: seasonality, estimated risks, and predictive factors. *J Parasit Dis.* 2019;**43**(2):198–208.

12. Spotts H, Walelign S, Tesfaye M, Desta K, Tsegaye A, Taye B. Concurrent infection of intestinal parasites and *Helicobacter pylori* among school-age children in Central Ethiopia. *Parasite Epidemiol Control.* 202;**11**:e00177.

13. Smith S, Fowora M, Pellicano R. Infections with *Helicobacter pylori* and challenges encountered in Africa. *World J Gastroenterol*. 2019;**25**(25):3183-3195. doi: 10.3748/wjg.v25.i25.3183. PMID: 31333310; PMCID: PMC6626727.

14. Pellicano R, Ménard A, Rizzetto M, Mégraud F. Helicobacter species and liver diseases: Association or causation? *Lancet Infect Dis*. 2008;**8**:254–260. doi: 10.1016/S1473-3099(08)70066-5.

15. de Korwin JD, Ianiro G, Gibiino G, Gasbarrini A. Helicobacter pylori infection and extragastric diseases in 2017. Helicobacter. 2017:22 Suppl 1. doi: 10.1111/hel.12411

16. Bhattacharjee S, Mejías-Luque R, Loffredo-Verde E, Toska A, Flossdorf M, Gerhard M, Prazeres da Costa C. Concomitant Infection of S. mansoni and H. pylori Promotes Promiscuity of Antigen-Experienced Cells and Primes the Liver for a Lower Fibrotic Response. *Cell Rep*. 2019;**28**(1):231-244.e5. doi: 10.1016/j.celrep.2019.05.108. PMID: 31269443.

17. Ifeanyi O, Adogu P. Prevalence of Helicobacter pylori and other intestinal parasites amongst duodenal and gastric ulcer patients at Imo state University Teaching Hospital, Orlu, south eastern Nigeria. *J Med Med Sci.* 2013;**4**:362–9.

18. Aniekwe O, Jolaiya T, Ajayi A, Adeleye IA, Gerhard M, Smith SI. Co-infection of Helicobacter pylori and intestinal parasites in children of selected low-income communities in Lagos State, Nigeria. *Parasitol Int.* 2024;**101**:102896. doi: 10.1016/j.parint.2024.102896.

19. Adewale B, Mafe MA, Mogaji HO, Balogun JB, Sulyman MA, Ajayi MB, Akande DO, Balogun EO. Urinary schistosomiasis and anemia among school-aged children from southwestern Nigeria. *Pathog Glob Health*. 2024;**118**(4):325-333. doi:

10.1080/20477724.2024.2322800. Epub 2024 Feb 28. PMID: 38419138; PMCID: PMC11234908.

20. Germani Y, Dauga C, Duval P, Huerre M, Levy M, Pialoux G, Sansonetti P, Grimont PA. Strategy for the detection of Helicobacter species by amplification of 16S rRNA genes and identification of H. felis in a human gastric biopsy. *Research in Microbiology*. 1997;**148**(4):315-26. doi: 10.1016/S0923-2508(97)81587-2.

21. Weiss J, Mecca J, Silva E, Gassner D. Comparison of PCR and other diagnostic techniques for detection of Helicobacter pylori infection in dyspeptic patients. *Journal of Clinical Microbiology.* 1994;**32**:1663–1668. doi: 10.1128/jcm.32.7.1663-1668.1994.

22. Wichmann D, Panning M, Quack T, Kramme S, Burchard GD, Grevelding C, Drosten C. Diagnosing schistosomiasis by detection of cell-free parasite DNA in human plasma. *PLoS Negl Trop Dis*. 2009;**3**(4):e422. doi:10.1371/journal.pntd.0000422

23. Hamburger J, Turetski T, Kapeller I, Deresiewicz R. Highly repeated short DNA sequences in the genome of Schistosoma mansoni recognized by a species-specific probe. *Mol Biochem Parasitol.* 1991;**44**(1):73-80. doi:10.1016/0166-6851(91)90222-r

24. Ikefuna A, Emerenini F, Nwolisa E, Iregbu F, Eke C. Prevalence and risk factors for *Helicobacter pylori* infection among children in Owerri, Nigeria. *Nigerian Journal of Clinical Practice*. 2021;24(8):1188-1193. doi:10.4103/njcp.njcp_687_20

25. Okoroiwu GIA, Okoroiwu IL, Ubosi NI, Sani NM. Sero-prevalence of and risk factors associated with *Helicobacter pylori* infections among individuals with peptic ulcer in Owerri, Imo State, Nigeria during 2020-2021. *African Journal of Clinical and Experimental Microbiology*. 2022;**23**(3):238-247. doi:10.4314/ajcem.v23i3.3

26. Ewa A, Abdulkadir Y, Ibrahim Y. Prevalence of Helicobacter Pylori Among Patients with Chronic Gastritis in Nigeria. *Clinical Medicine Research*. 2024;**13**(6):77-82. doi:10.11648/j.cmr.20241306.12

27. Smith S, Jolaiya T, Fowora M, Palamides P, Ngoka F, Bamidele M, Lesi O, Onyekwere C, Ugiagbe R, Agbo I, Ndububa D, Adekanle O, Adedeji A, Adeleye I, Harrison U. Clinical and Socio- Demographic Risk Factors for Acquisition of *Helicobacter pylori* Infection in Nigeria. *Asian Pacific Journal of Cancer Prevention*. 2018;**19**(7):1851-1857. doi: 10.22034/APJCP.2018.19.7.1851.

28. Holcombe C, Tsimiri S, Eldridge J, Jones DM. Prevalence of antibody to Helicobacter pylori in children in northern Nigeria. *Transactions of the Royal Society of Tropical Medicine and Hygiene*. 1993;**87**(1):19-21. doi:10.1016/0035-9203(93)90403-d

29. Smith SI, Ajayi A, Jolaiya TF, Essiet U. Prevalence, diagnosis and treatment of Helicobacter pylori infection in Nigeria. *Nigerian Journal of Gastroenterology and Hepatology*. 2022;**14**(1):2-10. doi:10.4103/njgh.njgh_10_22

30. Erturk E, Karaman U, Arici Y, Top S, Yolalan G. Factors Influencing Helicobacter Pylori Positivity in Children. Nigerian Journal of Clinical Practice. 2021;**24**(5):685-691. doi:10.4103/njcp.njcp_595_19

31. Chukwuma, O. M., Chukwuma, G. O., Manafa, P. O., Akulue, J. C., & Jeremiah, Z. A. Prevalence and possible risk factors for Helicobacter pylori seropositivity among peptic ulcerative individuals in Nnewi Nigeria. *BioMed Research Journal*. 2020; ***4***(1), 166-172.

32. Hooi JKY, Lai WY, Ng WK, et al. Global Prevalence of Helicobacter pylori Infection: Systematic Review and Meta-Analysis. *Gastroenterology*. 2017;**153**(2):420-429. doi:10.1053/j.gastro.2017.04.022

33. Singh K, Muddasiru D, Singh J. Current status of schistosomiasis in Sokoto, Nigeria. *Parasite Epidemiology and Control*. 2016;**1**(3):239-244. doi:10.1016/j.parepi.2016.08.003

34. Dawaki S, Al-Mekhlafi HM, Ithoi I, et al. The Menace of Schistosomiasis in Nigeria: Knowledge, Attitude, and Practices Regarding Schistosomiasis among Rural Communities in Kano State. *PLoS ONE*. 2015;**10**(11):e0143667. doi:10.1371/journal.pone.0143667

35. Adeniran AA, Mogaji HO, Aladesida AA, et al. Schistosomiasis, intestinal helminthiasis and nutritional status among preschool-aged children in sub-urban communities of Abeokuta, Southwest, Nigeria. *BMC Res Notes*. 2017;**10**(1). doi:10.1186/s13104-017-2973-2

36. Ojo JA, Adedokun SA, Akindele AA, et al. Prevalence of urogenital and intestinal schistosomiasis among school children in South-west Nigeria. *PLoS Negl Trop Dis*. 2021;**15**(7):e0009628. doi:10.1371/journal.pntd.0009628

37. Okeke OC, Akinwale OP, Ubachukwu PO, et al. Report of high prevalence of schistosome infection in Biomphalaria snails from a geographic area with no previous prevalence of human schistosomiasis in Nigeria. *Acta Tropica*. 2019;**210**:105326. doi:10.1016/j.actatropica.2019.105326

38. Ekpo UF, Oluwole AS, Abe EM, Etta HE, Olamiju F, Mafiana CF. Schistosomiasis in infants and pre-school-aged children in sub-Saharan Africa: implication for control. *Parasitology*. 2012;**139**(7):835-841. doi:10.1017/s0031182012000029

39. Eastham G, Fausnacht D, Becker MH, Gillen A, Moore W. Praziquantel resistance in schistosomes: a brief report. *Front Parasitol.* 2024;**3**:1471451. doi: 10.3389/fpara.2024.1471451. PMID: 39817170; PMCID: PMC11732111.

40. Olamiju F, Nebe OJ, Mogaji H, Marcus A, Amodu-Agbi P, Urude RO, Apake E, Olamiju O, Okoronkwo C, Achu I, Mpama O. Schistosomiasis outbreak during COVID-19 pandemic in Takum, Northeast Nigeria: Analysis of infection status and associated risk factors. *PLoS One*. 2022;**17**(1):e0262524. doi: 10.1371/journal.pone.0262524. PMID: 35061823; PMCID: PMC8782311.

41. Elshal MF, Elsayed IH, Kady IME, et al. Role of concurrent S. mansoni infection in H. pylori-associated gastritis: a flow cytometric DNA-analysis and oxyradicals correlations. *Clinica Chimica Acta*. 2004;**346**(2):191-198. doi:10.1016/j.cccn.2004.03.023

Click here to access/download
Supporting Information
S1 File.xlsx
