# Supplementary material for: Co-infections of Schistosoma mansoni and Helicobacter pylori in school-aged populations and implication for management and control practices in Niger State, Nigeria
Source: PLoS One. 2025 Dec 8;20(12):e0337423. doi: 10.1371/journal.pone.0337423 (PMC12685161; doi:10.1371/journal.pone.0337423)
Supplement: Attachment — Submitted filename: PONE-D-25-40863_R1 October 2025 PLOS ONE.pdf [file pone.0337423.s006.pdf]

PLOS ONE

Co-infections of Schistosoma mansoni and Helicobacter pylori in School-Aged Populations and implication for management and control practices in Niger State, Nigeria

--Manuscript Draft--

**Manuscript Number:**	PONE-D-25-40863R1
**Article Type:**	Research Article
**Full Title:**	Co-infections of Schistosoma mansoni and Helicobacter pylori in School-Aged Populations and implication for management and control practices in Niger State, Nigeria
**Short Title:**	Schistosoma mansoni and Helicobacter pylori in School-Aged Populations
**Corresponding Author:**	Hammed Oladeji Mogaji, Ph.D<br>Marian University<br>UNITED STATES OF AMERICA
**Keywords:**	Helicobacter pylori; Schistosoma mansoni; Co-infections; School-aged children; Chemotherapy; Nigerian; Elimination
**Abstract:**	Helicobacter pylori and Schistosoma mansoni are two important pathogens that infect the gastrointestinal tract, could be acquired earlier in childhood, and can remain asymptomatic for a long period. While S. mansoni is been controlled within routine primary healthcare program, there are no control guidelines for H. pylori. Here, we investigate the co-infection pattern of both pathogens to support improvement of management strategies. State. Fresh stool samples were obtained from 299 school-aged children between ages 6-17 years and analyzed using real-time and conventional polymerase chain reaction (PCR) for the direct detection of S. mansoni and H. pylori respectively. An overall prevalence of 19.7% (4.5% - 24.1%) was recorded for H. pylori, and 34.4% (0% - 42.1%) for S. mansoni, while co-infection of both was 7.0% (0% - 8.1%). Infections were significantly different across the study communities for S. mansoni (p = 0.00) when compared to H. pylori (p = 0.38). There were, however, no significant association between infection and gender (p > 0.05), with the following odds of infection for S. mansoni (OR=0.69, 95% CI: 0.42, 1.12), H. pylori (OR=1.33, 95% CI: 0.75, 2.36), and combination of both infections (OR=1.31, 95% CI: 0.83, 2.09). By age category, children below 14years were twice likely to be exposed to the combination of both infections; age 12-14 years (OR=1.9, 95% CI: 1.06, 3.44), and age 9-11 years (OR=1.96, 95% CI: 1.11, 3.48). But they were also less likely to be exposed to S. mansoni; age 12-14 years (OR=0.52, 95% CI: 0.27, 0.97), and age 9-11 years (OR=0.43, 95% CI: 0.23, 0.79). Our study highlights an unexpectedly high prevalence of S. mansoni, moderate prevalence of H. pylori, and very low coinfection prevalence for both. Our findings on S. mansoni are particularly concerning, suggesting potential challenges in control programs. It is therefore important to consider integrated interventions, including chemotherapy and improved water, sanitation, and hygiene (WASH) services.
**Order of Authors:**	Muinah Fowora
	Babatunde Adewale
	Hammed Mogaji, Ph.D
	Omolola Omolopo
	Balogun Daniel
	Abdulrahman Aladejana
	De'Broski R. Herbert
**Response to Reviewers:**	REVIEWER 1<br><br>The manuscript looks good. The study of the coinfections of Schistosoma mansoni and Helicobacter pylori is a good one and worthy of investigation as the two organisms cohabit gastrointestinal track. This has provided useful information and better

Powered by Editorial Manager® and ProduXion Manager® from Aries Systems Corporation

understanding on how they impact diseases etiology and this has great public health value and policy implications. The authors should clarify and reconcile the data presented in the manuscript. They should address all comments made on the manuscript.
Response; We are grateful for the very constructive and positive comments of the reviewer. We have now revised the manuscript taking into considerations all the reviewers comments and opinions

Comment 1: Full title is preferable.
Response: The title has been revised

Comment 2: It is not only MDA of praziquantel tablets that are being used for the elimination programme, rather other cross cutting approaches including Integrated Vector Management, WASH, Behavioural Change Communication are all part of the interventions that will facilitate elimination process.
Response: The abstract has also been revised accordingly. The new introductory lines now reads “Helicobacter pylori and Schistosoma mansoni are two important pathogens that infect the gastrointestinal tract, could be acquired earlier in childhood, and can remain asymptomatic for a long period. While S. mansoni is been controlled within routine primary healthcare program, there are no control guidelines for H. pylori. Here, we investigate the co-infection pattern of both pathogens to support improvement of management strategies..”

Comment 4: Does this refer to Niger basin or Niger State setting? kindly clarify.
Response: Yes, we have now revised the text to read “Niger State”

Comment 5: The findings for S. mansoni is quite disturbing as several rounds of MDAs have been implemented in Niger State. This calls to question the quality of intervention implemented all these years. The authors should have investigated the MDA history, therapeutic and geographical reach
Response: Yes, we agree absolutely with the reviewer, and noted this in our discussion. This observation is worth further investigation, and public health action.

Comments: Line 127-128: Why variation in the sample size
Response: We thank the reviewer for this important observation. This study is a sub-study, that follows a larger study where 410 children were recruited. However, for this sub-study only 73% consented to participate in this study. i.e. 100% compliance across all the communities except Monai (16.5% compliance). We have revised the sample-size estimation sub-section to provide more clarity.

Comments: Line 180-181: Kindly reconcile this number 299 with the earlier number of participants 410 under the smaple size
Response: We thank the reviewer for this important observation. This study is a sub-study, that follows a larger study where 410 children were recruited. However, for this sub-study only 73% consented to participate in this study. i.e. 100% compliance across all the communities except Monai (16.5% compliance). We have revised the sample-size estimation sub-section to provide more clarity.

Comments: Table 1, See comment above
Response: We thank the reviewer for this important observation. We have provided the response to this comment in our previous response.

Comments; Line 247-249: Kindly reconcile this statistics with the FMOH data base on schistosomiasis prevalence 2013-2017mappimg data. It is available with the National Programme and WHO ESPEN.
Response: We thank the reviewer for this important observation. We have revised the text to emphasize our comparison with research findings..

REVIEWER 2:
C1: Study overview
This study attempts to evaluate co-infections of Schistosoma mansoni and Helicobacter pylori among school-aged populations across five communities. Although, I find this initiative timely and important, the manuscript does not satisfactorily pitch the link between the two pathogens in the introduction, despite reporting findings that could

possibly inform future interventions. I hope my somewhat extensive comments do not discourage the authors and that they are received in the constructive and supportive spirit in which they are offered. For clarity, I have divided my comments into three categories, which I hope should help improve the overall presentation of your manuscript.
RESPONSE: We thank the reviewer for the comments, which have significantly improved the quality of our manuscript. We have carefully incorporated all the review suggestions made and hope you find the manuscript worthy of publication.

General comments:
1. It is not clear why these two pathogens are studied together. The manuscript does not precisely describe whether they share transmission pathway, immunological interaction etc. I expected to see a clearer rationale linking the topic and objective.
Response: We thank the reviewer for this comment. In our manuscript between lines 74-80, we describe the transmission pathway, clinical manifestations and speculations of altered disease pathology. We cited 6 references within this space. Further between lines 82-91, we further linked these lines with the rationale of generating evidence that improves the management of both diseases.

2. Also, I expected to read key findings that could inform changes in the management of both pathogens.
Response: We thank the reviewers for this comment. Following our analysis, we reported an unexpectedly high prevalence of S. mansoni, moderate prevalence of H. pylori, and very low coinfection prevalence for both. Our findings on S. mansoni is particularly concerning, suggesting potential challenges in control programs that needs investigation. While the co-infection rate was lower than expected, the findings still underscore the need for integrated interventions, including chemotherapy and improved water, sanitation, and hygiene (WASH) services, to address both infections effectively, especially among older children. These excerpts are currently in the abstract (lines; 32-43; results; discussion; conclusion (lines 271-277)

Comments 3. In the Methods section, the collection of data on water contact activities and parent's occupation was mentioned. However, these variables were neither included in the analysis nor reported in the Results and Discussion. Their omission, along with the absence of other important variables such as proxies for overcrowding and WASH indicators, somewhat weakens the manuscript, as such factors are crucial for guiding interventions.
Response: We appreciate the reviewer for this observation and clearly acknowledge this gap. We have revised these lines by deleting the variables that were not collected. Also, we added the context of the five study communities between lines 113-115 based on the previous study conducted amongst this population. The poor WASH access in the population still justifies the recommendations given, since our participants were drawn out (i.e., subsets) of that locality. Please see lines 124-126, and lines 133-137.

Comment 4. To emphasize the importance of this finding, consider noting in the Discussion (perhaps in the last paragraph) that schistosomiasis is classified as a Neglected Tropical Disease (NTD) according to the World Health Organization (WHO). This will highlight the importance of your work in identifying the locations and age groups with the highest prevalence odds of infection, thereby providing evidence to guide targeted interventions.

Response: We thank the reviewer for this comment. We have added a line to the last paragraph (please see line 264), and also introduced schistosomiasis as a NTD in the first line of our introduction.

Major comments

Comment 1. The introduction reads a bit underdeveloped. The literature review should be expanded to situate the study within existing knowledge. For example, lines 74-76 should more robustly explain the "similar risk" described. While both pathogens are WASH-related GIT, H. pylori is chiefly fecal-oral, oral-oral route often in childhood, while S. mansoni is via skin penetration by cercariae in contaminated water – both possessing different risk factors. As such, significant improvement is need in the introduction to satisfactorily present the study especially given what the study intend to

present.

Response: We appreciate the reviewer for this amazing comment, which was super useful. We have revised that paragraph to illustrate both the commonality and differences in their risk factors. Please see Line 75-79

Comments 2. Lines 234-236: Given that H. pylori infection is commonly reported during childhood and increases with age, the 1% seropositivity reported in Monai may be better explained by other unreported variables. Since these were omitted, the title should perhaps be revised to reflect the actual scope (demographic-related), rather than implying analyses that were not performed.
Response: We thank the reviewer for this comment. Considering the revisions we made, We have now provided the overall occupation, access to latrine and walking barefoot for these 5 communities studies. Based on this access to sanitation in Monai was 22%, and this resonates with the lines we have on Monai.

Comments 3. Consider starting your Discussion section with the presentation of your "principal findings", as this would enhance the quality of your manuscript. This way, you could move the first two sentences under Discussion to the latter part of the manuscript.
Response: We thank the reviewer for this comment. We have now moved the two sentences to the latter part of the manuscript

Comments 4. Lines 240 and 254: The text suggests a higher risk of co-infection among older children, which is inconsistent with both the study design and findings. The measure used in this study is OR, which captures "odds", and not "risk". Also, "Older children" is vague and not previously defined. The statement also overlooks the fact that no statistically significant evidence was found for the 15-17 age group. I recommend replacing "risk" with "chance". If the term "younger children" and "older children" are to be retained, age ranges should be defined in the Methods section. Otherwise, refer to the age ranges in the Discussion as they appear in the tables.

Response: We thank the reviewer for this comment. This is such an amazing observation. We have revised the text completely to replace risk with chance, and also to replace the younger or older children with the exact age categories as they appear in the tables. Please see Lines 240-266.

Minor comments

Comments 1. Keywords: It would be advisable to select keywords that do not overlap with the words in the title of the article, to improve discoverability during search.
Response: We thank the reviewer for these comments. This is well noted

Comments 2. Lines 24- 26: The statement appears awkward and incomplete, lacking logical flow between the two pathogens. The authors should attempt to establish explicit mechanistic link explaining why these pathogens are being co-investigated.
Response: We thank the reviewer for these comments. We have now revised the abstract to portray clarity.

Comments 3. Consider moving the ethical state to the final part of Methods, as is the common practice
Response: We thank the reviewer for these comments. We have now moved it.

Comments 4. The phrase "Niger area" is ambiguous, are you referring to Niger State, the River Niger basin, or another region? Please clarify.
Response: We thank the reviewer for these comments. We have now corrected this. We meant Niger State.

Comments: 5. Please justify the use of real-time PCR for S. mansoni and conventional PCR for H. pylori, especially since PCR detection of H. pylori is less common than antigen or urea breath testing.
Response: We thank the reviewer for these comments. We agree that using H. pylori detection is less common than antigen or urea breath testing. However, we already had primers and other reagents for the molecular detection in our laboratory. In the

face of limited funding for this work, we resulted to using PCR detection that was readily available in our laboratory. With regards to the use of qPCR for S. mansoni detection, studies have shown that RT-PCR was the most sensitive diagnostic method and gave the highest positive detection of Schistosoma species in stool specimen (Guegan et al. 2019). Hence, the use of RT-PCR for the detection of Schistosoma manosnii in this study.
Reference
Guegan H, Fillaux J, Charpentier E, Robert-Gangneux F, Chauvin P, Guemas E, et al. (2019) Real-time PCR for diagnosis of imported schistosomiasis. PLoS Negl Trop Dis 13(9): e0007711. https://doi.org/10.1371/journal.pntd.0007711

Comments; 6. The abstract overly descriptive. To enrich it, kindly incorporate a clear discussion of the key findings and potential explanations.

Response: We thank the reviewer for these comments. We have now revised the abstract to include these key findings as requested

Comments 7. Lines 101-103: Please specify what type of consent obtained and the database repository used.
Response: We thank the reviewer for this comment. Informed consent was obtained from parents/guardian and children. Children's assent was obtained verbally and documented via thumb print through an assent form in the presence of the parent or legal guardian who provided written consent. We used a password protected computer to create and assign unique ID

Comments 8. Lines 112 – 114: Please provide a reference to support this claim.
Response: We thank the reviewer for these comments. We have now provided the clear metrics and also he reference for this

Comments 9. There is a slight inconsistency in reported age of participants. E.g. the Methods states 6-16 yrs, but the Results report 6-17 yrs. Please address this discrepancy.
Response: We thank the reviewer for these comments. We have now revised the text to 6-17 years. key findings as requested

Comments: 10. The sample size justification is vague, lacking essential parameters such as expected prevalence and margin of error.
Response: We thank the reviewer for these comments. The sample size estimation followed WHO guidelines of recruiting 50 pupils per school/villages. And we adjusted this size by 60% to cater for non-enrolled school aged children. However, of the 410 children recruited for the larger project, only 73% consented to participate in this study. i.e. 100% compliance across all the communities except Monai (16.5% compliance).

Comments: 11. Line 140: Were participants instructed to provide stool samples at a specific time of the day, or was any sample acceptable? Given the collection period, how was sample quality and integrity ensured? Why was repeated sampling not considered, since egg shedding S. mansoni can be intermittent?
Response; Thanks for the comments. Yes, only fresh samples were retrieved, as detailed between lines 164-166. Samples were collected within a very limited time frame of 1hr, and the parents/legal guardians supervised the voiding process. The need for sample integrity was sufficiently explained to the parents and their children.

Comments 12. Line 142: Define "NIMR" at the first mention.
Response; Thanks for the comments. This has now been addressed

13. Kindly ensure that all scientific names are properly italicized following general taxonomy rules. There are sporadic occasions where this was not done in the manuscript (e.g. lines 147 and 148). Also, remove italics from "and" in "H. pylori and S. mansoni infections" at lines 193 and 205.
Response; Thanks for the comments. This has now been addressed

Comments 14. Kindly consider adding a minimal description of the variables included in the models is missing. What covariates were used? The supplementary materials do not include a data dictionary explaining each variable, and this information should still

Powered by Editorial Manager® and ProduXion Manager® from Aries Systems Corporation

	be included in the Methods since readers may not fully understand what is being tested.<br>Response; Thanks for the comments. This has now been addressed<br><br>Comments 15. Generally, reporting both p-values and confidence intervals together is somewhat redundant (e.g., lines 174 and 175, as well as throughout the Results section). If opting to continue with p-values, kindly standardize it throughout the manuscript, presenting all to three 3 significant digits.<br>Response; Thanks for the comments. We have standardized the p-value to 3 digits cross the text. However, we prefer to retain both the CI, to demonstrate precision around the estimate, and the p-value to demonstrate significance.<br><br>Comments 16. Line 183: The phrasing suggests a decreasing sequence, but this is not the case.<br>Response; Thanks for the comments. We have now addressed it<br><br>17. Add footnotes to tables defining all terms and abbreviations.<br>Response; Thanks for the comments. We have now addressed it<br><br>18. Line 220: It would be more appropriate and academically formal to use “Odds Ratio”.<br>Response; Thanks for the comments. We have now addressed it<br><br>19. Lines 224-225: Briefly specify what the “some evidence that might be important [...]” are<br>Response; Thanks for the comments. We have now addressed it. We moved this to the last paragraph as you earlier suggested and also, we rephrased it s’ our findings are useful in guiding more robust research and programmatic actions.<br><br>20. Line 227: “similar those”, please revise.<br>Response; Thanks for the comments. We have now addressed it<br>21. Lines 230-232: The justification here is unconvincing. Niger State is also in Northern Nigeria, and you have not conducted or presented any socio-environmental analysis to substantiate this claim.<br>Response; Thanks for the comments. Our comments here refer to the differences between our study and several other study in Nigeria that we cited. We were only trying to suggest the differences might be related to the diagnostic tool (as indeed we used PCR, which are more sensitive), and hygiene standard of the participants or sites.<br>22. While the use of ORs in cross-sectional studies is common, this being a prevalence study, results may risk overestimation. In this case, Prevalence Ratios (PRs) might provide a more accurate measure of association. However, I do not discourage the use of ORs, but it would be worthwhile to mention this as a limitation.<br>Response; Thanks for the comments., and we appreciate the reviewers concern on this.<br><br>23. Line 260: What exactly was “previously predicted”? No hypotheses were explicitly stated earlier.<br>Response; Thanks for the comments. We have now addressed it by removing that<br><br>24. Per journal policy, please deposit the script and supporting data in an open repository.<br>Response; Thanks for the comments. We have provided the supporting data.
**Additional Information:**	
**Question**	**Response**
**Financial Disclosure**<br><br>Enter a financial disclosure statement that describes the sources of funding for the work included in this submission. Review the submission guidelines for detailed requirements. View published research	Yes

articles from *PLOS ONE* for specific examples.<br><br>This statement is required for submission and **will appear in the published article** if the submission is accepted. Please make sure it is accurate.<br><br>**Funded studies**<br>Enter a statement with the following details:<br>• Initials of the authors who received each award<br>• Grant numbers awarded to each author<br>• The full name of each funder<br>• URL of each funder website<br>• Did the sponsors or funders play any role in the study design, data collection and analysis, decision to publish, or preparation of the manuscript?<br><br>Did you receive funding for this work?	
Please add funding details.<br>as follow-up to "**Financial Disclosure**<br><br>Enter a financial disclosure statement that describes the sources of funding for the work included in this submission. Review the submission guidelines for detailed requirements. View published research articles from *PLOS ONE* for specific examples.<br><br>This statement is required for submission and **will appear in the published article** if the submission is accepted. Please make sure it is accurate.	This work received partial support from the Nigerian Institute of Medical Research Grant for studies on Neglected Tropical Diseases [Grant number 0000134].

**Funded studies**<br>Enter a statement with the following details:<br>• Initials of the authors who received each award<br>• Grant numbers awarded to each author<br>• The full name of each funder<br>• URL of each funder website<br>• Did the sponsors or funders play any role in the study design, data collection and analysis, decision to publish, or preparation of the manuscript?<br><br>Did you receive funding for this work?"	
Please select the country of your main research funder (please select carefully as in some cases this is used in fee calculation).<br>as follow-up to "**Financial Disclosure**<br><br>Enter a financial disclosure statement that describes the sources of funding for the work included in this submission. Review the submission guidelines for detailed requirements. View published research articles from *PLOS ONE* for specific examples.<br><br>This statement is required for submission and **will appear in the published article** if the submission is accepted. Please make sure it is accurate.<br><br>**Funded studies**<br>Enter a statement with the following details:<br>• Initials of the authors who received each award<br>• Grant numbers awarded to each author<br>• The full name of each funder<br>• URL of each funder website<br>• Did the sponsors or funders play any role in the study design, data collection and analysis, decision to publish, or preparation of the manuscript?<br><br>Did you receive funding for this work?"	NIGERIA - NG
**Competing Interests**	The authors have declared that no competing interests exist.

Use the instructions below to enter a competing interest statement for this submission. On behalf of all authors, disclose any competing interests that could be perceived to bias this work—acknowledging all financial support and any other relevant financial or non-financial competing interests.<br><br>This statement is required for submission and **will appear in the published article** if the submission is accepted. Please make sure it is accurate and that any funding sources listed in your Funding Information later in the submission form are also declared in your Financial Disclosure statement.<br><br>View published research articles from *PLOS ONE* for specific examples.<br><br>**NO authors have competing interests**<br><br>Enter: *The authors have declared that no competing interests exist.*<br><br>**Authors with competing interests**<br><br>Enter competing interest details beginning with this statement:<br><br>*I have read the journal's policy and the authors of this manuscript have the following competing interests: [insert competing interests here]*<br><br>* typeset	
**Ethics Statement**<br><br>Enter an ethics statement for this submission. This statement is required if the study involved:<br><br>• Human participants<br>• Human specimens or tissue<br>• Vertebrate animals or cephalopods<br>• Vertebrate embryos or tissues	The study received approval from the Nigerian Institute of Medical Research ethical review board (IRB/18/042). Informed consent was obtained from parents/guardian and children. Children’s assent was obtained verbally and documented via thumb print through an assent form in the presence of the parent or legal guardian who provided written consent. Unique identifiers and a password-protected database were also used to ensure anonymity and confidentiality through the study procedures. The study procedures were implemented following the ethical standards of the Helsinki Declaration (1964, amended most recently in 2008) of the World Medical Association.

• Field research<br><br>Write "N/A" if the submission does not require an ethics statement.<br><br>General guidance is provided below. Consult the submission guidelines for detailed instructions. **Make sure that all information entered here is included in the Methods section of the manuscript.**	

Powered by Editorial Manager® and ProduXion Manager® from Aries Systems Corporation

**Format for specific study types**<br><br>**Human Subject Research (involving human participants and/or tissue)**<br>• Give the name of the institutional review board or ethics committee that approved the study<br>• Include the approval number and/or a statement indicating approval of this research<br>• Indicate the form of consent obtained (written/oral) or the reason that consent was not obtained (e.g. the data were analyzed anonymously)<br><br>**Animal Research (involving vertebrate animals, embryos or tissues)**<br>• Provide the name of the Institutional Animal Care and Use Committee (IACUC) or other relevant ethics board that reviewed the study protocol, and indicate whether they approved this research or granted a formal waiver of ethical approval<br>• Include an approval number if one was obtained<br>• If the study involved *non-human primates*, add *additional details* about animal welfare and steps taken to ameliorate suffering<br>• If anesthesia, euthanasia, or any kind of animal sacrifice is part of the study, include briefly which substances and/or methods were applied<br><br>**Field Research**<br><br>Include the following details if this study involves the collection of plant, animal, or other materials from a natural setting:<br>• Field permit number<br>• Name of the institution or relevant body that granted permission	
**Data Availability**<br><br>Authors are required to make all data underlying the findings described fully available, without restriction, and from the time of publication. PLOS allows rare exceptions to address legal and ethical concerns. See the PLOS Data Policy and FAQ for detailed information.	Yes - all data are fully available without restriction

A Data Availability Statement describing where the data can be found is required at submission. Your answers to this question constitute the Data Availability Statement and **will be published in the article**, if accepted.<br><br>**Important:** Stating 'data available on request from the author' is not sufficient. If your data are only available upon request, select 'No' for the first question and explain your exceptional situation in the text box.<br><br>Do the authors confirm that all data underlying the findings described in their manuscript are fully available without restriction?	
**Describe where the data may be found in full sentences. If you are copying our sample text, replace any instances of XXX with the appropriate details.**<br><br>• If the data are **held or will be held in a public repository**, include URLs, accession numbers or DOIs. If this information will only be available after acceptance, indicate this by ticking the box below. For example: *All XXX files are available from the XXX database (accession number(s) XXX, XXX.*).<br>• If the data are all contained **within the manuscript and/or Supporting Information files**, enter the following: *All relevant data are within the manuscript and its Supporting Information files.*<br>• If neither of these applies but you are able to provide **details of access elsewhere**, with or without limitations, please do so. For example:<br><br>*Data cannot be shared publicly because of [XXX]. Data are available from the XXX Institutional Data Access / Ethics Committee (contact via XXX) for researchers who meet the criteria for access to confidential data.*<br><br>*The data underlying the results presented in the study are available from (include the name of the third party*	The datasets used and/or analyzed are available as supplementary files

and contact information or URL).<br>• This text is appropriate if the data are owned by a third party and authors do not have permission to share the data.<br><br>* typeset	
Additional data availability information:	

Powered by Editorial Manager® and ProduXion Manager® from Aries Systems Corporation

Manuscript Click here to access/download;Manuscript;Clean manuscript file.docx

Co-infections of *Schistosoma mansoni* and *Helicobacter pylori* in School-Aged Populations and implication for management and control practices in Niger State, Nigeria.

Muinah Fowora[1], Babatunde Adewale[2], Hammed Mogaji [2,3,4]*, Omolola Omolopo[1], Balogun Daniel[2], Abdulrahman Aladejana[5], and De'Broski R. Herbert[6]

[1]Molecular Biology and Biotechnology Department, Nigerian Institute of Medical Research Yaba, Lagos Nigeria.

[2]Public Health and Epidemiology Department, Nigerian Institute of Medical Research, Yaba, Lagos Nigeria.

[3]Public Health Unit, Department of Applied and Behavioral Sciences, College of Arts and Sciences, Marian University, United States of America

[4]Department of Research and Development, Mission To Save The Helpless, Lagos State, Nigeria

[5]Department of Animal and Environmental Biology, Federal University Oye-Ekiti, Ekiti State, Nigeria

[6]Department of Microbiology and Immunology, Tulane University School of Medicine, New Orleans, LA, United States of America

Corresponding author: *mogajihammed@gmail.com

Keywords: *Schistosoma mansoni*, *H. pylori*, Co-infections, School-aged children, Nigeria

Abstract

Helicobacter pylori and *Schistosoma mansoni* are two important pathogens that infect the gastrointestinal tract, could be acquired earlier in childhood, and can remain asymptomatic for a long period. While *S. mansoni* is been controlled within routine primary healthcare program, there are no control guidelines for *H. pylori.* Here, we investigate the co-infection pattern of both pathogens to support improvement of management strategies. State. Fresh stool samples were obtained from 299 school-aged children between ages 6-17 years and analyzed using real-time and conventional polymerase chain reaction (PCR) for the direct detection of *S. mansoni* and *H. pylori* respectively. An overall prevalence of 19.7% (4.5% - 24.1%) was recorded for *H. pylori*, and 34.4% (0% - 42.1%) for *S. mansoni,* while co-infection of both was 7.0% (0% - 8.1%). Infections were significantly different across the study communities for *S. mansoni* ($p = 0.00$) when compared to *H. pylori* ($p = 0.38$). There were, however, no significant association between infection and gender ($p > 0.05$), with the following odds of infection for *S. mansoni* (OR=0.69, 95% CI: 0.42, 1.12), *H. pylori* (OR=1.33, 95% CI: 0.75, 2.36), and combination of both infections (OR=1.31, 95% CI: 0.83, 2.09). By age category, children below 14years were twice likely to be exposed to the combination of both infections; age 12-14 years (OR=1.9, 95% CI: 1.06, 3.44), and age 9-11 years (OR=1.96, 95% CI: 1.11, 3.48). But they were also less likely to be exposed to *S. mansoni*; age 12-14 years (OR=0.52, 95% CI: 0.27, 0.97), and age 9-11 years (OR=0.43, 95% CI: 0.23, 0.79). Our study highlights an unexpectedly high prevalence of *S. mansoni*, moderate prevalence of H. *pylori*, and very low coinfection prevalence for both. Our findings on *S. mansoni* are particularly concerning, suggesting potential challenges in control programs. It is therefore important to consider integrated interventions, including chemotherapy and improved water, sanitation, and hygiene (WASH) services.

Keywords: *Helicobacter pylori, S. mansoni*, Chemotherapy, Nigerian, Elimination

Introduction

Schistosomiasis is one of the most prevalent neglected tropical diseases in the world with more than 200 million persons affected worldwide across 78 countries, mostly in areas where access to water, sanitation and hygiene facilities are lacking [1-4]. The disease is caused by water-borne trematode parasites of the genus Schistosoma, with two major species of *Schistosoma*; the first is the *S. haematobium* which inhabits the vesicular and pelvic venous plexus of the bladder and causes urogenital schistosomiasis and the second is *S. mansoni* which is more often in the inferior mesenteric veins draining the large intestine and causes intestinal schistosomiasis [2-4]. Schistosomiasis is a focal disease, with urogenital schistosomiasis been more prevalent, and perhaps more studied in endemic settings compared to, intestinal schistosomiasis [5]. Intestinal schistosomiasis has severe acute complications associated with the intestine - abdominal pain, diarrhea, blood in the stool, and in more severe cases, could lead to chronic enlargement of the liver and spleen, a condition known as hepatosplenomegaly [5-8]. The pathologies are worsened among children because of the developing immunity, with already established evidence on anemia, stunting, protein-energy malnutrition, school absenteeism and reduced cognition [9-10].

People infected with *S. mansoni* may be infected with *Helicobacter pylori* (*H. pylori*), a Gram-negative bacterium that also colonizes the gastrointestinal tract [11,12]. *H. pylori* is the most common human bacterium, and causative agent of gastritis, peptic ulcer disease, mucosa

associated lymphoid tissue lymphoma and gastric cancer (GC) [13]. This bacterium infects 50% of the world's population, with about 80% of African population affected [13]. The risk factors for *H. pylori* acquisition have been reported to be mainly due to overcrowding, to have infected siblings or parents and to unsafe water sources [13]. Both *H. pylori* and *S. mansoni* share similar risk as transmission is intertwined with poor socio-ecological and economic factors, primarily lack or sub-optimal access to water and sanitation facilities, and poor hygiene practices [7,13]. However, *H. pylori* are chiefly transmitted via the fecal-oral or oral-oral route often in childhood, unlike *S. mansoni* which requires cercariae skin penetration [7,8,13]. Nevertheless, both pathogens infect the gastrointestinal tract, could be acquired earlier in childhood, can remain asymptomatic for a long period [7,8,13]. In regions where co-infections occur, there are overlapping clinical manifestations, with extra-gastroduodenal manifestations such as chronic liver diseases and iron-deficiency anemia [14-15]. There are also speculations of altered disease pathology, either as synergistic or antagonistic interactions, which may influence treatment and clinical outcomes, and susceptibility to other diseases [16].

Efforts targeted at controlling schistosomiasis have been ongoing for over a decade in Africa, and other endemic settings with routine diagnosis and administration of large scale praziquantel medications [1-3], there are currently no guidelines targeted at diagnosing or controlling *H. pylori* infections within routine primary health care programs [13]. In Nigeria, the most populous country in Africa, several studies have reported the co-infection of *H. pylori* with intestinal parasites and soil transmitted helminths and its associated burden [17, 18], but studies on co-infections of *H. pylori* and *S. mansoni* are scarce. This evidence is necessary to support improvement of management strategies. This study therefore reports the prevalence and associated risk factors of

H. pylori and *S. mansoni* co-infection among school-aged population across five study communities in Niger State of Nigeria.

Materials and Methods

Study area, design and selection of sampling sites

This study was conducted between 1st and 30th of April 2019, as part of a larger project, with the area and selection procedure previously described [19]. Briefly, five communities (Monai, Tamanai, Koro, Musarwa, and Yuna) perceived to be at risk of water-borne infections were purposively selected based on proximity to Kainji Dam in Borgu LGA of Niger state. Majority of the populations in these communities engage in farming (36.3%), fishing (12.9%), and trading (7.1%) [19]. In terms of access to WASH, only 31% have access to improved sources of water and latrine facilities, and about 47% walk barefooted [19]. We employed a cross-sectional approach to administer pretested questionnaires and obtain fresh stool sample collection from school-aged children between ages 6-16 years.

Sample Size Determination and Selection of Study Participants

Participants recruited in this study were subset of those recruited in the larger study [19]. Four hundred and ten pupils living in communities close to Kainji Dam were enlisted following their informed consent in the larger project. The sample size was calculated followed WHO guidelines for recruiting a minimum of 50 pupils (above age 5) per school during helminthiasis survey [11]. The sample size was adjusted by 60% in each of the communities to cater for non-enrolled school-aged children. As such, a minimum of 400 children was targeted across the five selected

communities. However, the recruitment of participants was in variant with the estimated sample size for some communities because of number of eligible participants who presented themselves for the study. Children within the age range of 6-17 years, who had not received any anti-helminth treatment within the last 6 months, were recruited from the communities for the study. Of the 410 children enrolled in the larger study, only 22/133 from Monai, 79/79 from Tamanai, 124/124 from Koro, 36/36 from Musawa, and 38/38 from Yuna, consented to participate in this study. Information on the age and sex of children was collected using simple field forms.

Questionnaire Administration

Each pupil was allotted a unique code of six digits representing community, class and serial numbers. Demographic data such as name, age, sex were recorded using a simple field. The assigned participants ID were inscribed on both consent/assent form, field form and stool specimen bottle. The field form was designed in the English language but administered in Hausa language.

Collection of Stool Samples

Participants were provided with one sterile specimen bottle pre-labelled with their unique identification number, an applicator stick, a plain sheet of paper, and tissue paper to clean their anus. Participants were instructed to defecate on the plain sheet of paper and use the applicator stick to transfer a fresh portion into the specimen bottle. Specimen bottles were retrieved within 1 h of distribution, but collection was between 10 h and 14 h. All collected stool samples were sorted and transported in iceboxes for processing within 2 hours of collection to the Nigerian Institute of Medical Research (NIMR) outstation laboratory located in Kainji.

Laboratory processing of stool samples

The endemicity was classified based on the aggregated prevalence in each community. For H. pylori identification, stored stool sample in ice-boxes were transported to the Molecular Biology and Biotechnology Department of the Nigerian Institute of Medical Research. Genomic DNA was extracted and purified from the stool samples using NIMR Biotech stool DNA extraction kit in accordance with the manufacturer's instructions. An initial Polymerase Chain Reaction (PCR) was carried out using *Helicobacter* species specific primers as previously described [20]. All samples positive for the presence of *Helicobacter* species were further analyzed with a second PCR targeting the 16S rRNA gene of *H pylori*. The second PCR reaction was carried out using the primer set Hpy-F (5′-CGC ACC TGC TGG AAC ATT AC-3′) and Hpy-R (5′-CGT TAG CTG CAT TAC TGG AGA-3′) as previously described [21]. All PCR fragments were separated on a 2% agarose gel and visualized after ethidium bromide staining. A 138-bp fragment confirmed the presence of *H. pylori* in the stool sample.

A real time-PCR was used to detect cell-free *S. mansoni* in the DNA samples. The reaction was carried out using the set of primers and probes that amplifies a 121 bp tandem repeat sequence of *S. mansoni* strain SM 1-7 (GenBank accession number: M61098) as previously described [22, 23]. The primer and probe sequence were Sm FW 5′-CCG ACC AAC CGT TCT ATG A-3′; Sm RV 5′-CAC GC TCT CG C AAA TAA TCT AAA-3′; Sm probe 5′-[FAM] TCG TTG TAT CTC CGA AAC CAC TGG ACG [(BHQ1])-3′ all synthesized by Biomers GMBH, Germany.

Data Management and Analysis

Data obtained from this study were imported and analyzed in R. software version 4.3.2 (S1 File). Descriptive statistics, including frequencies and percentages, were used to describe the prevalence and co-infection patterns, and tested association across communities using chi-square statistics. Significant associations were established when $p < 0.05$. Furthermore, we performed three univariate logistic regression model each between single (*H. pylori, S. mansoni*), co-infection (*H pylori* and *S. mansoni*) as response variables, and demographic factor (sex and age) as covariates Regression estimates were reported in the form of odds ratio (OR) and 95% confidence interval (CI). The significance level was established as $p < 0.05$.

Ethical Statement and Considerations

The study received approval from the Nigerian Institute of Medical Research ethical review board (IRB/18/042). Informed consent was obtained from parents/guardian and children. Children's assent was obtained verbally and documented via thumb print through an assent form in the presence of the parent or legal guardian who provided written consent. Unique identifiers and a password-protected database were also used to ensure anonymity and confidentiality through the study procedures. The study procedures were implemented following the ethical standards of the Helsinki Declaration (1964, amended most recently in 2008) of the World Medical Association.

Results

Demographic Characteristics of Study Participants

Table 1 presents the age and sex distribution of the 299 participants recruited for the study from five communities: Monai (n=22, 7.4%), Tamani (n=79, 26.4%), Koro (n=124, 41.5%), Musawa (n=36, 12.0%), and Yuna (n=38, 12.7%). Male participants (n=174, 58.2%) were more than females (n=125, 41.8%). The age group distribution showed that most participants were aged 9-11 years (n=102, 34.1%), followed by 6-8 years (n=96, 32.1%), 12-14 years (n=90, 30.1%), and 15-17 years (n=11, 3.7%). Significant differences were observed in the age and sex distribution across the communities (p = 0.00). Monai presented the highest proportion of participants aged 6-8 years (21, 95.5%), while Yuna had the highest proportion in the 12-14 years category (25, 65.8%). Males were significantly more represented in Yuna (34, 89.5%) compared to other communities (p = 0.00).

Table 1: Sex and age characteristics of the study population

		**Communities**					
	**N = 299**	**Monai (N = 22)**	**Tamani (N = 79)**	**Koro (N = 124)**	**Musawa (N = 36)**	**Yuna (N = 38)**	**p-value**
**age, N (%)**							0.00
6-8yrs	96 (32.1%)	21 (95.5%)	5 (6.3%)	41 (33.1%)	22 (61.1%)	7 (18.4%)	
9-11yrs	102 (34.1%)	1 (4.5%)	43 (54.4%)	38 (30.6%)	14 (38.9%)	6 (15.8%)	
12-14yrs	90 (30.1%)	0 (0.0%)	26 (32.9%)	39 (31.5%)	0 (0.0%)	25 (65.8%)	
15-17yrs	11 (3.7%)	0 (0.0%)	5 (6.3%)	6 (4.8%)	0 (0.0%)	0 (0.0%)	
**sex, N (%)**							0.00
Female	125 (41.8%)	8 (36.4%)	41 (51.9%)	56 (45.2%)	16 (44.4%)	4 (10.5%)	
Male	174 (58.2%)	14 (63.6%)	38 (48.1%)	68 (54.8%)	20 (55.6%)	34 (89.5%)	

N: Number of participants

Prevalence of *Helicobacter pylori* and *Schistosoma mansoni* Infections

Table 2 summarizes the prevalence of *H. pylori and S. mansoni* infections among the participants. An overall prevalence of 19.7% was recorded for *Helicobacter pylori* across the communities. Tamani (24.1%) had the highest prevalence, while Monai (4.5%) had the lowest prevalence. However, there were no statistically significant differences in *H. pylori* prevalence across the communities (p = 0.38). In contrast, *S. mansoni* had a significantly higher overall prevalence of 34.4%. Yuna (42.1%), closely followed by Koro (41.1%) had the highest prevalence across communities, while Monai reported no cases (0.0%). The differences in *S. mansoni* prevalence across communities were statistically significant (p =0.00). Participants were further categorized based on their infection status: no infection (52.8%), mono-infection (40.1%), or co-infection (7.0%). Co-infection rates were highest in Koro (8.1%), followed by Yuna (7.9%), while Monai had (0%) coinfection rate. These results were statistically different among the communities (p = 0.00).

Table 2: Prevalence of *H. pylori and S. mansoni* infection among the study population

	N = 299	Monai (N = 22)	Tamani (N = 79)	Koro (N = 124)	Musawa (N = 36)	Yuna (N = 38)	p-value
H. pylori							0.38
Positive	59 (19.7%)	1 (4.5%)	19 (24.1%)	24 (19.4%)	7 (19.4%)	8 (21.1%)	
Negative	240 (80.3%)	21 (95.5%)	60 (75.9%)	100 (80.6%)	29 (80.6%)	30 (78.9%)	
S. mansoni							0.00
Positive	103 (34.4%)	0 (0.0%)	29 (36.7%)	51 (41.1%)	7 (19.4%)	16 (42.1%)	
Negative	196 (65.6%)	22 (100.0%)	50 (63.3%)	73 (58.9%)	29 (80.6%)	22 (57.9%)	
**Category**							0.00
None	158 (52.8%)	21 (95.5%)	37 (46.8%)	59 (47.6%)	24 (66.7%)	17 (44.7%)	
Mono	120 (40.1%)	1 (4.5%)	36 (45.6%)	55 (44.4%)	10 (27.8%)	18 (47.4%)	
Double	21 (7.0%)	0 (0.0%)	6 (7.6%)	10 (8.1%)	2 (5.6%)	3 (7.9%)	

N: Number of participants

Association Between Infections and Demographic Factors

Table 3 shows the association between infections and demographic variables. Compared to females, males had non-significant associations with *H. pylori* (OR: 1.33, 95% CI: 0.75–2.36, p =0.33), *S. mansoni* (OR: 0.69, 95% CI: 0.42–1.12, p =0.14) and co-infection (OR: 1.31, 95% CI: 0.83–2.09, p =0.25). By age, participants aged 9-11 and 12-14years had significant lower odds of infection with *S. mansoni* (OR: 0.43, 95% CI: 0.23–0.79, p = 0.01) and (OR: 0.52, 95% CI: 0.27–0.97, p = 0.04), respectively when compared to those aged 6-8 years. However, the odds of co-infection with *H. pylori and S. mansoni* were significantly higher among participants aged 9-11 years (OR: 1.96, 95% CI: 1.11–3.48, p = 0.02) and 12-14 years (OR: 1.90, 95% CI: 1.06–3.44, p = 0.03) compared to those aged 6-8 years.

Table 3: Association between infections and participants demographic

	**Pathogens**					
	H. pylori		***S. mansoni***		**Co-infection**	
	OR (95% CI)	p-value	OR (95% CI)	p-value	OR (95% CI)	p-value
**sex, N (%)**						
Female	1		1		1	
Male	1.33 (0.75, 2.36)	0.33	0.69 (0.42, 1.12)	0.14	1.31 (0.83, 2.09)	0.25
**age, N(%)**						
6-8yrs	1		1		1	
9-11yrs	0.77(0.37, 1.58)	0.48	0.43(0.23, 0.79)	0.01	1.96 (1.11, 3.48)	0.02
12-14yrs	0.75 (0.35, 1.56)	0.44	0.52 (0.27, 0.97)	0.04	1.9 (1.06, 3.44)	0.03
15-17yrs	0.53 (0.14, 2.63)	0.39	0.84 (0.22, 4.07)	0.81	1.45 (0.39, 5.36)	0.56

N: Number of participants; OR: Odds Ratio; CI: Confidence Interval

Discussion

Foremost, the overall prevalence recorded for *H. pylori* was 19.7%, which is similar to those reported in Owerri (20%) [24], but much lower than those reported in Imo State (72%) [25], Kano (70% and 38%) [26,27]. The northern part of Nigeria has been known to be heavily impacted [28,29], owing to increased risk associated with overcrowding and limited access to water and sanitation facilities [24,25]. This report, however, deviates from this notion, and disparities could be attributed to differences in diagnostic tools, sampling sites and study participants, or in fact, the hygiene standards of the latter two factors.

We observed *H. pylori* prevalence was particularly low in Monai compared to other communities which could suggest a difference in community-level sanitation practices [19], or health interventions, or maybe linked to age, as about 96% of those in Monai were below age 8years. However, we couldn't find any association between mono-infections with *H. pylori* based on age. We presume our limited sample-size and covariate frame might have impacted the identification of any meaningful association. However, our regression model (for combined infection) further emphasized increased chance of infection in children aged 12-14years compared to those between age 9-11years. Previous studies have reported in consonance that the chance of infections is increased among older participants [25,26,30,31]. A possible explanation for the higher prevalence of infection among children aged 12-14 years is that exposures and reinfection are likely to persist in areas where WASH services are limited and socio-economic status are low, both conditions which reflect the nature of our study location [19, 30,31,32].

For *S. mansoni*, an overall prevalence of 34.4% (19.4% - 42.1%) was recorded, this is quite high considering previous research findings in Nigeria — 2.93% in Sokoto [33], 8.93% in Kano [34], 3.6% in Ogun [35], and 9% in Osun [36]. This presents a source of concern for control programs with questions on persistent transmission considering environmental suitability and ineffective control program [37,38], or on a more worrisome note, the efficacy of praziquantel on *S. mansoni* [39]. Infections also shared similar patterns with *H. pylori* mono infection and with literature [35,38,40], as children aged 12-14 years were more infected, and none of the children aged 6-8 years in Monai were infected. The odds of infection, even though lower in younger children were seen to progressively increase in magnitude as children age. These findings are logical, as infection with schistosomes is related to behavioral patterns, most especially water contact practices which are more common among older children due to increased liberty to engage in risky behaviors, such as swimming, bathing and fetching in river bodies infested with *Biomphalaria* snail species [35,38,40].

Our observation of a 7.0% co-infection rate is less concerning. However, considering the efforts targeted controlling schistosomiasis over the last decade, the high prevalence of *S. mansoni* alone was surprising and raises significant public health concerns, underscoring the need for enhanced chemotherapy and improved water, sanitation, and hygiene (WASH) services. Reports of synergistic and antagonistic immunological interactions between *S. mansoni* and *H. pylori* [16,41] prompt further questions about whether these pathogens might exhibit antagonistic relationships at the ecological or behavioral levels, potentially influencing transmission dynamics. This could explain the lower-than-expected co-infection prevalence observed in this study. While our findings are not robust enough to conclusively address this hypothesis, the question merits further

investigation to better understand the interplay between these two pathogens in co-endemic settings. In this study, we have reported the prevalence of co-infection with *H. pylori and S. mansoni* infections among school-aged children in five communities in Northern Nigeria. Our findings are useful in guiding more robust research and programmatic actions.

Conclusion

Our study highlights an unexpectedly high prevalence of *S. mansoni*, moderate prevalence of H. *pylori*, and very low coinfection prevalence for both. Our findings on *S. mansoni* is particularly concerning, suggesting potential challenges in control programs, that needs investigation. While the co-infection rate was lower than expected, the findings still underscore the need for integrated interventions, including chemotherapy and improved water, sanitation, and hygiene (WASH) services, to address both infections effectively, especially among older children.

Supporting Information File

S1 File: Dataset

Declarations

Authors' contribution

MF, BA, and HM conceptualized the study and prepared the protocol, DH approved the protocol. BA and HM coordinated field surveys. BD, AA were involved in data collection. MF and OO carried out the molecular analysis. HM performed statistical analysis, and MF and HM prepared the first draft of the manuscript. All authors contributed to the development of the final manuscript

and approved its submission.

Conflict of Interest

The authors declare that the research was conducted in the absence of any commercial or financial relationships that could be construed as a potential conflict of interest.

Availability of data and materials

The datasets used and/or analyzed are available as supplementary files

Acknowledgements

We are grateful to the community leaders across the study communities, and all the health workers who facilitated the implementation process.

References

1. World Health Organization (WHO). Ending the neglect to attain the Sustainable Development Goals: a road map for neglected tropical diseases 2021–2030. Geneva: World Health Organization; 2020. Licence: CC BY-NC-SA 3.0 IGO.

2. World Health Organization (WHO). Schistosomiasis. 2021. https://www.who.int/news-room/fact-sheets/detail/schistosomiasis. Accessed 20 September 2021.

3. World Health Organization (WHO). Helminth control in school-age children: a guide for managers of control programmes. 2021. https://www.who.int/neglected_diseases/resources/9789241548267/en/. Accessed 20 September 2021.

4. Midzi N, Mduluza T, Chimbar MJ, Tshuma C, Charimar L, et al. Distribution of schistosomiasis and soil transmitted helminthiasis in Zimbabwe: towards a national plan of action for control and elimination. *PLoS Negl Trop Dis*. 2014;**8**, e3014. pmid:25121489

5. McManus, D.P., Dunne, D.W., Sacko, M. *et al.* Schistosomiasis. *Nat Rev Dis Primers* **4**, 13 (2018). https://doi.org/10.1038/s41572-018-0013-8

6. Centre for Disease Control (CDC). Schistosomiasis. Laboratory identification of parasites of public health concern. https://www.cdc.gov/dpdx/schistosomiasis/index.html. Accessed 20 September 2021.

7. Costain AH, MacDonald AS, Smits HH. Corrigendum: Schistosome Egg Migration: Mechanisms, Pathogenesis and Host Immune Responses. *Front. Immunol*. 2019;**10**: 749. pmid:31031753

8. Jose RL. Acute schistosomiasis mansoni: revisited and reconsidered. Mem Inst Oswaldo Cruz, Rio de Janeiro. 2010;**105**(4):422–435. pmid:20721485

9. Guerrant RL, Leite AM, Pinkerton R, Medeiros PH, Cavalcante PA et al. Biomarkers of Environmental Enteropathy, Inflammation, Stunting, and Impaired Growth in Children in Northeast Brazil. *PLoS ONE*. 2016;**11**(9):e0158772. pmid:27690129; PubMed Central PMCID: PMC5045163.

10. Osakunor DNM, Woolhouse MEJ, Mutapi F. Paediatric schistosomiasis: What we know and what we need to know. *PLoS Negl Trop Dis*. 2018;**12**(2): e0006144. pmid:29420537

11. Montresor, A, Crompton, DWT, Hall A, Bundy DAP, Savioli L. Guidelines for the Evaluation of soil-Transmitted Hel-Minthiasis and Schistosomiasis at Community Level: A guide for Managers of Control Programmes. World Health Organization. 1998. Available online: **https://apps.who.int/iris/handle/10665/63821** (accessed on 15 January 2023).

12. Spotts H, Walelign S, Tesfaye M, Desta K, Tsegaye A, Taye B. Concurrent infection of intestinal parasites and *Helicobacter pylori* among school-age children in Central Ethiopia. *Parasite Epidemiol Control.* 202;**11**:e00177.

13. Smith S, Fowora M, Pellicano R. Infections with *Helicobacter pylori* and challenges encountered in Africa. *World J Gastroenterol*. 2019;**25**(25):3183-3195. doi: 10.3748/wjg.v25.i25.3183. PMID: 31333310; PMCID: PMC6626727.

14. Pellicano R, Ménard A, Rizzetto M, Mégraud F. Helicobacter species and liver diseases: Association or causation? *Lancet Infect Dis*. 2008;**8**:254–260. doi: 10.1016/S1473-3099(08)70066-5.

15. de Korwin JD, Ianiro G, Gibiino G, Gasbarrini A. Helicobacter pylori infection and extragastric diseases in 2017. Helicobacter. 2017:22 Suppl 1. doi: 10.1111/hel.12411

16. Bhattacharjee S, Mejías-Luque R, Loffredo-Verde E, Toska A, Flossdorf M, Gerhard M, Prazeres da Costa C. Concomitant Infection of S. mansoni and H. pylori Promotes Promiscuity of Antigen-Experienced Cells and Primes the Liver for a Lower Fibrotic Response. *Cell Rep*. 2019;**28**(1):231-244.e5. doi: 10.1016/j.celrep.2019.05.108. PMID: 31269443.

17. Ifeanyi O, Adogu P. Prevalence of Helicobacter pylori and other intestinal parasites amongst duodenal and gastric ulcer patients at Imo state University Teaching Hospital, Orlu, south eastern Nigeria. *J Med Med Sci.* 2013;**4**:362–9.

18. Aniekwe O, Jolaiya T, Ajayi A, Adeleye IA, Gerhard M, Smith SI. Co-infection of Helicobacter pylori and intestinal parasites in children of selected low-income communities in Lagos State, Nigeria. *Parasitol Int.* 2024;**101**:102896. doi: 10.1016/j.parint.2024.102896.

19. Adewale B, Mafe MA, Mogaji HO, Balogun JB, Sulyman MA, Ajayi MB, Akande DO, Balogun EO. Urinary schistosomiasis and anemia among school-aged children from southwestern Nigeria. *Pathog Glob Health*. 2024;**118**(4):325-333. doi: 10.1080/20477724.2024.2322800. Epub 2024 Feb 28. PMID: 38419138; PMCID: PMC11234908.

20. Germani Y, Dauga C, Duval P, Huerre M, Levy M, Pialoux G, Sansonetti P, Grimont PA. Strategy for the detection of Helicobacter species by amplification of 16S rRNA genes and identification of H. felis in a human gastric biopsy. *Research in Microbiology*. 1997;**148**(4):315-26. doi: 10.1016/S0923-2508(97)81587-2.

21. Weiss J, Mecca J, Silva E, Gassner D. Comparison of PCR and other diagnostic techniques for detection of Helicobacter pylori infection in dyspeptic patients. *Journal of Clinical Microbiology.* 1994;**32**:1663–1668. doi: 10.1128/jcm.32.7.1663-1668.1994.

22. Wichmann D, Panning M, Quack T, Kramme S, Burchard GD, Grevelding C, Drosten C. Diagnosing schistosomiasis by detection of cell-free parasite DNA in human plasma. *PLoS Negl Trop Dis*. 2009;**3**(4):e422. doi:10.1371/journal.pntd.0000422

23. Hamburger J, Turetski T, Kapeller I, Deresiewicz R. Highly repeated short DNA sequences in the genome of Schistosoma mansoni recognized by a species-specific probe. *Mol Biochem Parasitol.* 1991;**44**(1):73-80. doi:10.1016/0166-6851(91)90222-r

24. Ikefuna A, Emerenini F, Nwolisa E, Iregbu F, Eke C. Prevalence and risk factors for *Helicobacter pylori* infection among children in Owerri, Nigeria. *Nigerian Journal of Clinical Practice*. 2021;24(8):1188-1193. doi:10.4103/njcp.njcp_687_20

25. Okoroiwu GIA, Okoroiwu IL, Ubosi NI, Sani NM. Sero-prevalence of and risk factors associated with *Helicobacter pylori* infections among individuals with peptic ulcer in Owerri, Imo State, Nigeria during 2020-2021. *African Journal of Clinical and Experimental Microbiology*. 2022;**23**(3):238-247. doi:10.4314/ajcem.v23i3.3

26. Ewa A, Abdulkadir Y, Ibrahim Y. Prevalence of Helicobacter Pylori Among Patients with Chronic Gastritis in Nigeria. *Clinical Medicine Research*. 2024;**13**(6):77-82. doi:10.11648/j.cmr.20241306.12

27. Smith S, Jolaiya T, Fowora M, Palamides P, Ngoka F, Bamidele M, Lesi O, Onyekwere C, Ugiagbe R, Agbo I, Ndububa D, Adekanle O, Adedeji A, Adeleye I, Harrison U. Clinical and Socio- Demographic Risk Factors for Acquisition of *Helicobacter pylori* Infection in

Nigeria. *Asian Pacific Journal of Cancer Prevention*. 2018;**19**(7):1851-1857. doi: 10.22034/APJCP.2018.19.7.1851.

28. Holcombe C, Tsimiri S, Eldridge J, Jones DM. Prevalence of antibody to Helicobacter pylori in children in northern Nigeria. *Transactions of the Royal Society of Tropical Medicine and Hygiene*. 1993;**87**(1):19-21. doi:10.1016/0035-9203(93)90403-d

29. Smith SI, Ajayi A, Jolaiya TF, Essiet U. Prevalence, diagnosis and treatment of Helicobacter pylori infection in Nigeria. *Nigerian Journal of Gastroenterology and Hepatology*. 2022;**14**(1):2-10. doi:10.4103/njgh.njgh_10_22

30. Erturk E, Karaman U, Arici Y, Top S, Yolalan G. Factors Influencing Helicobacter Pylori Positivity in Children. Nigerian Journal of Clinical Practice. 2021;**24**(5):685-691. doi:10.4103/njcp.njcp_595_19

31. Chukwuma, O. M., Chukwuma, G. O., Manafa, P. O., Akulue, J. C., & Jeremiah, Z. A. Prevalence and possible risk factors for Helicobacter pylori seropositivity among peptic ulcerative individuals in Nnewi Nigeria. *BioMed Research Journal*. 2020; ***4***(1), 166-172.

32. Hooi JKY, Lai WY, Ng WK, et al. Global Prevalence of Helicobacter pylori Infection: Systematic Review and Meta-Analysis. *Gastroenterology*. 2017;**153**(2):420-429. doi:10.1053/j.gastro.2017.04.022

33. Singh K, Muddasiru D, Singh J. Current status of schistosomiasis in Sokoto, Nigeria. *Parasite Epidemiology and Control*. 2016;**1**(3):239-244. doi:10.1016/j.parepi.2016.08.003

34. Dawaki S, Al-Mekhlafi HM, Ithoi I, et al. The Menace of Schistosomiasis in Nigeria: Knowledge, Attitude, and Practices Regarding Schistosomiasis among Rural Communities in Kano State. *PLoS ONE*. 2015;**10**(11):e0143667. doi:10.1371/journal.pone.0143667

35. Adeniran AA, Mogaji HO, Aladesida AA, et al. Schistosomiasis, intestinal helminthiasis and nutritional status among preschool-aged children in sub-urban communities of Abeokuta, Southwest, Nigeria. *BMC Res Notes*. 2017;**10**(1). doi:10.1186/s13104-017-2973-2

36. Ojo JA, Adedokun SA, Akindele AA, et al. Prevalence of urogenital and intestinal schistosomiasis among school children in South-west Nigeria. *PLoS Negl Trop Dis*. 2021;**15**(7):e0009628. doi:10.1371/journal.pntd.0009628

37. Okeke OC, Akinwale OP, Ubachukwu PO, et al. Report of high prevalence of schistosome infection in Biomphalaria snails from a geographic area with no previous prevalence of human schistosomiasis in Nigeria. *Acta Tropica*. 2019;**210**:105326. doi:10.1016/j.actatropica.2019.105326

38. Ekpo UF, Oluwole AS, Abe EM, Etta HE, Olamiju F, Mafiana CF. Schistosomiasis in infants and pre-school-aged children in sub-Saharan Africa: implication for control. *Parasitology*. 2012;**139**(7):835-841. doi:10.1017/s0031182012000029

39. Eastham G, Fausnacht D, Becker MH, Gillen A, Moore W. Praziquantel resistance in schistosomes: a brief report. *Front Parasitol.* 2024;**3**:1471451. doi: 10.3389/fpara.2024.1471451. PMID: 39817170; PMCID: PMC11732111.

40. Olamiju F, Nebe OJ, Mogaji H, Marcus A, Amodu-Agbi P, Urude RO, Apake E, Olamiju O, Okoronkwo C, Achu I, Mpama O. Schistosomiasis outbreak during COVID-19 pandemic in Takum, Northeast Nigeria: Analysis of infection status and associated risk factors. *PLoS One*. 2022;**17**(1):e0262524. doi: 10.1371/journal.pone.0262524. PMID: 35061823; PMCID: PMC8782311.

41. Elshal MF, Elsayed IH, Kady IME, et al. Role of concurrent S. mansoni infection in H. pylori-associated gastritis: a flow cytometric DNA-analysis and oxyradicals correlations. *Clinica Chimica Acta*. 2004;**346**(2):191-198. doi:10.1016/j.cccn.2004.03.023

Click here to access/download
Supporting Information
S1 File.xlsx

Revised Manuscript with Track Changes

Co-infections of *Schistosoma mansoni* and *Helicobacter pylori* in School-Aged Populations and implication for management and control practices in Niger State, Nigeria.

Muinah Fowora[1], Babatunde Adewale[2], Hammed Mogaji [2,3,4]*, Omolola Omolopo[1], Balogun Daniel[2], Abdulrahman Aladejana[5], and De'Broski R. Herbert[6]

[1]*Molecular Biology and Biotechnology Department, Nigerian Institute of Medical Research Yaba, Lagos Nigeria*.

[2]Public Health and Epidemiology Department, Nigerian Institute of Medical Research, Yaba, Lagos Nigeria.

[3]Public Health Unit, Department of Applied and Behavioral Sciences, College of Arts and Sciences, Marian University, United States of America

[4]Department of Research and Development, Mission To Safe The Helpless, Lagos State, Nigeria

[5]Department of Animal and Environmental Biology, Federal University Oye-Ekiti, Ekiti State, Nigeria

[6]Department of Microbiology and Immunology, Tulane University School of Medicine, New Orleans, LA, United States of America

Corresponding author: *mogajihammed@gmail.com

Keywords: *Schistosoma mansoni*, *H. pylori*, ~~Association~~Co-infections, School-aged children, Nigeria

Abstract

Helicobacter pylori and *Schistosoma mansoni* are two important pathogens that infect the gastrointestinal tract, could be acquired earlier in childhood, and can remain asymptomatic for a long period. While *S. mansoni* is been controlled within routine primary healthcare program, there are no control guidelines for *H. pylori*. Here, we investigate the co-infection pattern of both pathogens to support improvement of management strategies. ~~*Schistosoma mansoni* is a chronic human intestinal parasite that is targeted for elimination through mass drug administration of praziquantel. *Helicobacter pylori*, a pathogenic bacterium that also colonizes the gastrointestinal tract, but the understanding of how co-infections impact disease pathology is poorly understood. Hence, we conducted a cross-sectional study to investigate the co-infections of both pathogens among school-aged population sampled across five study communities in Niger area~~ State~~of Nigeria~~. Fresh stool samples were obtained from 299 school-aged children between ages 6-17 years and analyzed using real-time and conventional polymerase chain reaction (PCR) for the direct detection of *S. mansoni* and *H. pylori* respectively. An overall prevalence of 19.7% (4.5% - 24.1%) was recorded for *H. pylori*, and 34.4% (0% - 42.1%) for *S. mansoni,* while co-infection of both was 7.0% (0% - 8.1%). Infections were significantly different across the study communities for *S. mansoni* (p = 0.00~~<0.05~~) when compared to *H. pylori* (p ~~>~~ = 0.38). There were, however, no significant association between infection and gender (p > 0.05), with the following odds of infection for *S. mansoni* (OR=0.69, 95% CI: 0.42, 1.12), *H. pylori* (OR=1.33, 95% CI: 0.75, 2.36), and combination of both infections (OR=1.31, 95% CI: 0.83, 2.09). By age category, children below 14years were twice likely to be exposed to the combination of both infections; age 12-14 years (OR=1.9, 95% CI: 1.06, 3.44), and age 9-11 years (OR=1.96, 95% CI: 1.11, 3.48). But they were also less likely to be exposed to *S. mansoni*; age 12-14 years (OR=0.52, 95% CI: 0.27, 0.97),

Formatted: Line spacing: Double, Tab stops: Not at 0.5" + 1" + 1.5" + 2" + 2.5" + 3" + 3.5" + 4" + 4.5" + 5" + 5.5" + 6.5"

Formatted: Font: Italic

Formatted: Font: Italic

and age 9-11 years (OR=0.43, 95% CI: 0.23, 0.79). Our study highlights an unexpectedly high prevalence of *S. mansoni*, moderate prevalence of H. *pylori*, and very low coinfection prevalence for both. Our findings on *S. mansoni* are particularly concerning, suggesting potential challenges in control programs. It is therefore important to consider ~~While the co-infection rate is low, the findings on *S. mansoni* are particularly concerning, which underscore the need for i~~integrated interventions, including chemotherapy and improved water, sanitation, and hygiene (WASH) services.

Keywords: *Helicobacter pylori, S. mansoni*, Chemotherapy, Nigerian, Elimination

Introduction

Schistosomiasis is one of the most prevalent neglected tropical diseases in the world with more than 200 million persons affected worldwide across 78 countries, mostly in areas where access to water, sanitation and hygiene facilities are lacking [1-4]. The disease is caused by water-borne trematode parasites of the genus Schistosoma, with two major species of *Schistosoma*; the first is the *S. haematobium* which inhabits the vesicular and pelvic venous plexus of the bladder and causes urogenital schistosomiasis and the second is *S. mansoni* which is more often in the inferior mesenteric veins draining the large intestine and causes intestinal schistosomiasis [2-4]. Schistosomiasis is a focal disease, with urogenital schistosomiasis been more prevalent, and perhaps more studied in endemic settings compared to, intestinal schistosomiasis [5]. Intestinal

schistosomiasis has severe acute complications associated with the intestine - abdominal pain, diarrhea, blood in the stool, and in more severe cases, could lead to chronic enlargement of the liver and spleen, a condition known as hepatosplenomegaly [5-8]. The pathologies are worsened among children because of the developing immunity, with already established evidence on anemia, stunting, protein-energy malnutrition, school absenteeism and reduced cognition [9-10].

People infected with *S. mansoni* may be infected with *Helicobacter pylori* (*H. pylori*), a Gram-negative bacterium that also colonizes the gastrointestinal tract [11,12]. *H. pylori* is the most common human bacterium, and causative agent of gastritis, peptic ulcer disease, mucosa associated lymphoid tissue lymphoma and gastric cancer (GC) [13]. This bacterium infects 50% of the world's population, with about 80% of African population affected [13]. The risk factors for *H. pylori* acquisition have been reported to be mainly due to overcrowding, to have infected siblings or ~~parent~~parents and to unsafe water sources [13]. Both *H. pylori* and *S. mansoni* share similar risk ~~been~~ as transmission is intertwined with poor socio-ecological and economic factors, primarily ~~that~~lack or sub-optimal access to water and sanitation facilities, and poor hygiene practices [7,13]. However, *H. pylori* are chiefly transmitted via the fecal-oral or oral-oral route often in childhood, unlike *S. mansoni*, which requires cercariae skin penetration [7,8,13]. Nevertheless, ~~they~~ both pathogens infect the gastrointestinal tract, could be acquired earlier in childhood, can remain asymptomatic for a long period [7,8,13]. In regions where co-infections occur, there are overlapping clinical manifestations, with extra-gastroduodenal manifestations such as chronic liver diseases and iron-deficiency anemia [14-15]. There are also speculations of altered disease pathology, either as synergistic or antagonistic interactions, which may influence treatment and clinical outcomes, and susceptibility to other diseases [16].

Formatted: Font: (Default) Times New Roman, 12 pt

Formatted: Font: (Default) Times New Roman, 12 pt, Italic

Formatted: Font: (Default) Times New Roman, 12 pt

Efforts targeted at controlling schistosomiasis have been ongoing for over a decade in Africa, and other endemic settings with routine diagnosis and administration of large scale praziquantel medications [1-3], there are currently no guidelines targeted at diagnosing or controlling *H. pylori* infections within routine primary health care programs [13]. In Nigeria, the most populous country in Africa, several studies have reported the co-infection of *H. pylori* with intestinal parasites and soil transmitted ~~helminthes~~helminths and its associated burden [17, 18], but studies on co-infections of *H. pylori* and *S. mansoni* are scarce. This evidence is necessary to support improvement of management strategies. This study therefore reports the prevalence and associated risk factors of *H. pylori* and *S. mansoni* co-infection among school-aged population across five study communities in Niger State of Nigeria.

Materials and Methods

~~**Ethical Statement and Considerations**~~

~~The study received approval from the Nigerian Institute of Medical Research ethical review board (IRB/18/042). Informed consent was obtained from parents/guardian and children. Children's assent was obtained verbally and documented through an assent form in the presence of the parent or legal guardian. Unique identifiers and a password-protected database were also used to ensure anonymity and confidentiality through the study procedures. The study procedures were~~

implemented following the ethical standards of the Helsinki Declaration (1964, amended most recently in 2008) of the World Medical Association.

Study area, design and selection of sampling sites

This study was conducted between 1st and 30th of April 2019, as part of a larger project, with the area and selection procedure previously described [19]. Briefly, five communities (Monai, Tamanai, Koro, Musarwa, and Yuna) perceived to be at risk of water-borne infections were purposively selected based on proximity to Kainji Dam in Borgu LGA of Niger state. Majority of the populations in these communities engage in farming (36.3%), fishing (12.9%), and trading (7.1%) [19]. In terms of access to WASH, only , and there 31% have access is limited access to improved sources of water and latrine facilities, and about 47% walk barefooted [19]. We employed a cross-sectional approach to administer pretested questionnaires and obtain fresh stool sample collection from school-aged children between ages 6-16 years.

Sample Size Determination and Selection of Study Participants

Participants recruited in this study were subset of those recruited in the larger study [19]. Four hundred and ten pupils living in close proximity communities close to Kainji Dam were enlisted following their informed consent in the larger project. The sample size was calculated using the followed WHO guidelines for recruiting a minimum of 50 pupils (above age 5) per school during helminthiasis survey [11]. The sample size was adjusted by 60% in each of the communities to

cater for non-enrolled school-aged children. As such, a minimum of 400 children was targeted across the five selected communities. However, the recruitment of participants was in variant with the estimated sample size for some communities because of number of eligible participants who presented themselves for the study.sample size estimate from a National survey with a 95% confidence interval[11]. Children within the age range of 6-17 years, who had not received any anti-helminth treatment within the last 6 months, were recruited from the communities for the study. Of the 410 children enrolled in the larger study, only 22/133 from Monai, 79/79 from Tamanai, 124/124 from Koro, 36/36 from Musawa, and 38/38 from Yuna, consented to participate in this study. Information on the age and sex , sex and water contact activities of children was collected using simple field formsstructured questionnaires. . Children within the age range of 5-16 years, who had not received any anti-helminth treatment within the last 6 months, were recruited from the communities for the study. Summarily, 410 children were enrolled in the larger study, only 22/133 from Monai, 79/79 from Tamanai, 124/124 from Koro, 36/36 from Musawa, and 38/38 from Yuna, participated in this study.

Questionnaire Administration

Each Using semi-structured questionnaires each pupil was allotted a unique code of six digits representing community, class and serial numbers. D while demographic data such as includes the name, age, sex, and parents' occupation were recorded using a simple field. The and assigned participants ID were inscribed on both consent/assent form, field form and stool specimen bottle. The field form was designed in the English language but administered in Hausa language.

Collection of Stool Samples

Participants were provided with one sterile specimen bottle pre-labelled with their unique identification number, an applicator stick, a plain sheet of paper, and tissue paper to clean their anus. Participants were instructed to defecate on the plain sheet of paper and use the applicator stick to transfer a fresh portion into the specimen bottle. Specimen bottles were retrieved within 1 h of distribution, but collection was between 10 h and 14 h. All collected stool samples were sorted and transported in iceboxes for processing within 2 hours of collection to the Nigerian Institute of Medical Research (NIMR) outstation laboratory located in Kainji.

Laboratory processing of stool samples

The endemicity was classified based on the aggregated prevalence in each community. For H. pylori identification, stored stool sample in ice-boxes were transported to the Molecular Biology and Biotechnology Department of the Nigerian Institute of Medical Research. Genomic DNA was extracted and purified from the stool samples using NIMR Biotech stool DNA extraction kit in accordance with the manufacturer's instructions. An initial Polymerase Chain Reaction (PCR) was carried out using *Helicobacter* species specific primers as previously described [20]. All samples positive for the presence of *Helicobacter* species were further analyzed with a second PCR targeting the 16S rRNA gene of *H pylori*. The second PCR reaction was carried out using the primer set Hpy-F (5′-CGC ACC TGC TGG AAC ATT AC-3′) and Hpy-R (5′-CGT TAG CTG CAT TAC TGG AGA-3′) as previously described [21]. All PCR fragments were separated on a 2% agarose gel and visualized after ethidium bromide staining. A 138-bp fragment confirmed the presence of *H. pylori* in the stool sample.

A real time-PCR was used to detect cell-free *S. mansoni* in the DNA samples. The reaction was carried out using the set of primers and probes that amplifies a 121 bp tandem repeat sequence of *S. mansoni* strain SM 1-7 (GenBank accession number: M61098) as previously described [22, 23]. The primer and probe sequence were Sm FW 5′-CCG ACC AAC CGT TCT ATG A-3′; Sm RV 5′-CAC GC TCT CG C AAA TAA TCT AAA-3′; Sm probe 5′-[FAM] TCG TTG TAT CTC CGA AAC CAC TGG ACG [(BHQ1])-3′ all synthesized by Biomers GMBH, Germany.

Data Management and Analysis

Data obtained from this study were imported and ~~analysed~~analyzed in R. software version 4.3.2 (S1 File). Descriptive statistics, including frequencies and percentages, were used to describe the prevalence and co-infection patterns, and tested association across communities using chi-square statistics. Significant associations were established when $p < 0.05$. Furthermore, we performed three univariate logistic regression model each between single (*H. pylori, S. mansoni*), co-infection (*H pylori* and *S. mansoni*) as response variables, and demographic factor (sex and age) as covariates~~.~~ Regression estimates were reported in the form of odds ratio~~s~~ (OR) and 95% confidence interval~~s~~ (CI). The significance level was established as $p < 0.05$.

Ethical Statement and Considerations

The study received approval from the Nigerian Institute of Medical Research ethical review board (IRB/18/042). Informed consent was obtained from parents/guardian and children. Children’s assent was obtained verbally and documented via thumb print through an assent form in the

presence of the parent or legal guardian who provided written consent. Unique identifiers and a password-protected database were also used to ensure anonymity and confidentiality through the study procedures. The study procedures were implemented following the ethical standards of the Helsinki Declaration (1964, amended most recently in 2008) of the World Medical Association.

Results

Demographic Characteristics of Study Participants

Table 1 presents the age and sex distribution of the 299 participants recruited for the study from five communities: Monai (n=22, 7.4%), Tamani (n=79, 26.4%), Koro (n=124, 41.5%), Musawa (n=36, 12.0%), and Yuna (n=38, 12.7%). Male participants (n=174, 58.2%) were more than females (n=125, 41.8%). The age group distribution showed that the majority ofmost participants were aged 6-8 years (n=96, 32.1%), followed by 9-11 years (n=102, 34.1%), followed by 6-8 years (n=96, 32.1%), 12-14 years (n=90, 30.1%), and 15-17 years (n=11, 3.7%). Significant differences were observed in the age and sex distribution across the communities (p = 0.00< 0.001). Monai presented the highest proportion of participants aged 6-8 years (21, 95.5%), while Yuna had the highest proportion in the 12-14 years category (25, 65.8%). Males were significantly more represented in Yuna (34, 89.5%) compared to other communities (p <= 0.001).

Table 1: Sex and age characteristics of the study population

		Communities					
	**N = 299**	**Monai (N = 22)**	**Tamani (N = 79)**	**Koro (N = 124)**	**Musawa (N = 36)**	**Yuna (N = 38)**	**p-value**
**age, N~~n~~ (%)**							~~<~~0.00~~1~~
6-8yrs	96 (32.1%)	21 (95.5%)	5 (6.3%)	41 (33.1%)	22 (61.1%)	7 (18.4%)	
9-11yrs	102 (34.1%)	1 (4.5%)	43 (54.4%)	38 (30.6%)	14 (38.9%)	6 (15.8%)	
12-14yrs	90 (30.1%)	0 (0.0%)	26 (32.9%)	39 (31.5%)	0 (0.0%)	25 (65.8%)	
15-17yrs	11 (3.7%)	0 (0.0%)	5 (6.3%)	6 (4.8%)	0 (0.0%)	0 (0.0%)	
**sex, ~~n~~ N (%)**							0~~<0~~.00~~1~~
Female	125 (41.8%)	8 (36.4%)	41 (51.9%)	56 (45.2%)	16 (44.4%)	4 (10.5%)	
Male	174 (58.2%)	14 (63.6%)	38 (48.1%)	68 (54.8%)	20 (55.6%)	34 (89.5%)	

N: Number of participants

Prevalence of *Helicobacter pylori* and *Schistosoma mansoni* Infections

Table 2 summarizes the prevalence of *H. pylori and S. mansoni* infections among the participants. An overall prevalence of 19.7% was recorded for *Helicobacter pylori* across the communities. Tamani (24.1%) had the highest prevalence, while Monai (4.5%) had the lowest prevalence. However, there were no statistically significant differences in *H. pylori* prevalence across the communities (p = 0.38). In contrast, *S. mansoni* had a significantly higher overall prevalence of 34.4%. Yuna (42.1%), closely followed by Koro (41.1%) had the highest prevalence across communities, while Monai reported no cases (0.0%). The differences in *S. mansoni* prevalence across communities were statistically significant (p ~~<~~ =0.000~~0.001~~). Participants were further categorized based on their infection status: no infection (52.8%), mono-infection (40.1%), or co-infection (7.0%). Co-infection rates were highest in Koro (8.1%), followed by Yuna (7.9%), while

Monai had (0%) coinfection rate. These results were statistically different among the communities (p = 0.004).

Table 2: Prevalence of *H. pylori and S. mansoni* infection among the study population

	N = 299	Monai (N = 22)	Tamani (N = 79)	Koro (N = 124)	Musawa (N = 36)	Yuna (N = 38)	p-value
H. pylori							0.38
Positive	59 (19.7%)	1 (4.5%)	19 (24.1%)	24 (19.4%)	7 (19.4%)	8 (21.1%)	
Negative	240 (80.3%)	21 (95.5%)	60 (75.9%)	100 (80.6%)	29 (80.6%)	30 (78.9%)	
S. mansoni							<0.001
Positive	103 (34.4%)	0 (0.0%)	29 (36.7%)	51 (41.1%)	7 (19.4%)	16 (42.1%)	
Negative	196 (65.6%)	22 (100.0%)	50 (63.3%)	73 (58.9%)	29 (80.6%)	22 (57.9%)	
**Category**							0.004
None	158 (52.8%)	21 (95.5%)	37 (46.8%)	59 (47.6%)	24 (66.7%)	17 (44.7%)	
Mono	120 (40.1%)	1 (4.5%)	36 (45.6%)	55 (44.4%)	10 (27.8%)	18 (47.4%)	
Double	21 (7.0%)	0 (0.0%)	6 (7.6%)	10 (8.1%)	2 (5.6%)	3 (7.9%)	

N: Number of participants

Association Between Infections and Demographic Factors

Table 3 shows the association between infections and demographic variables. Compared to females, males had non-significant associations with *H. pylori* (OR: 1.33, 95% CI: 0.75–2.36, p =0.33), *S. mansoni* (OR: 0.69, 95% CI: 0.42–1.12, p =0.14) and co-infection (OR: 1.31, 95% CI: 0.83–2.09, p =0.25). By age, participants aged 9-11 and 12-14years had significant lower odds of infection with *S. mansoni* (OR: 0.43, 95% CI: 0.23–0.79, p = 0.01) and (OR: 0.52, 95% CI: 0.27–0.97, p = 0.04), respectively when compared to those aged 6-8 years. However, the odds of co-infection with *H. pylori and S. mansoni* were significantly higher among participants aged 9-11 years (OR: 1.96, 95% CI: 1.11–3.48, p = 0.02) and 12-14 years (OR: 1.90, 95% CI: 1.06–3.44, p = 0.03) compared to those aged 6-8 years.

Table 3: Association between infections and participants demographic

	Pathogens					
	H. pylori		***S. mansoni***		**Co-infection**	
	OR (95% CI)	p-value	OR (95% CI)	p-value	OR (95% CI)	p-value
**sex, ~~n~~ N (%)**						
Female	1		1		1	
Male	1.33 (0.75, 2.36)	0.33	0.69 (0.42, 1.12)	0.14	1.31 (0.83, 2.09)	0.25
**age, N~~n~~ (%)**						
6-8yrs	1		1		1	
9-11yrs	0.77(0.37, 1.58)	0.48	0.43(0.23, 0.79)	0.01	1.96 (1.11, 3.48)	0.02
12-14yrs	0.75 (0.35, 1.56)	0.44	0.52 (0.27, 0.97)	0.04	1.9 (1.06, 3.44)	0.03
15-17yrs	0.53 (0.14, 2.63)	0.39	0.84 (0.22, 4.07)	0.81	1.45 (0.39, 5.36)	0.56

N: Number of participants; OR: Odds Ratio~~s~~; CI: Confidence Interval

-Discussion

Formatted: Left, Line spacing: Multiple 1.08 li

~~In this study, we have reported the prevalence of co-infection with *H. pylori and S. mansoni* infections among school-aged children in five communities in Northern Nigeria. Our findings have provided some form of evidence that might be important to guide future directions for more robust research and programmatic actions.~~ Foremost, the overall prevalence recorded for *H. pylori* was 19.7%, which is similar to those reported in Owerri (20%) [24], but much lower than those reported in Imo State (72%) [25], Kano (70% and 38%) [26,27]. The northern part of Nigeria has been known to be heavily impacted [28,29], owing to increased risk associated with overcrowding and limited access to water and sanitation facilities [24,25]. This report however, deviates from this notion, and disparities could be attributed to differences in diagnostic tools, sampling sites and study participants, or in fact, the hygiene standards of the latter two factors.~~.~~

We observed *H. pylori* prevalence was particularly low in Monai compared to other communities which could suggest a difference in community-level sanitation practices [19], or health interventions, or maybe linked to age, as about 96% of those in Monai were below age 8years. However, we couldn't find any association between mono-infections with *H. pylori* based on age. We presume our limited sample-size and covariate frame might have impacted the identification of any meaningful association. However, our regression model (for combined infection) further emphasized increased chance ~~risk~~ of infection in ~~older~~ children aged 12-14years compared to ~~younger~~ those between ~~ones~~age 9-11years. Previous studies have reported in consonance that the chance ~~risk~~ of infections is increased among older participants [25,26,30,31]. A possible explanation for the higher prevalence ~~rate~~ of infection among children aged 12-14 years~~older children~~ is that exposures and reinfection are ~~likely going~~likely to persist in areas where WASH

services are limited and socio-economic status are low, both ~~condition~~conditions which reflect the nature of our study location [19, 30,31,32].

For *S. mansoni*, an overall prevalence of 34.4% (19.4% - 42.1%) was recorded, this is quite high considering previous research findings in Nigeria — 2.93% in Sokoto [33], 8.93% in Kano [34], 3.6% in Ogun [35], and 9% in Osun [36]. This presents a source of concern for control programs with questions on persistent transmission considering environmental suitability and ineffective control program [37,38], or on a more worrisome note, the efficacy of praziquantel on *S. mansoni* [39]. Infections also shared similar ~~pattern~~patterns with *H. pylori* mono infection and with ~~literature [~~literature [35,38,40], as children aged 12-14 years ~~older children~~ were more infected, and none of the ~~younger~~ children aged 6-8 years in Monai were infected. The odds of infection, even though lower in younger children were seen to progressively increase in magnitude as children age. These findings are logical, as infection with schistosomes is related to behavioral patterns, most especially water contact practices which are more common among older children due to increased liberty to engage in risky behaviors, such as swimming, bathing and fetching in river bodies infested with *B~~b~~iomphalaria* snail species [35,38,40].

Formatted: Font: Italic

Our observation of a 7.0% co-infection rate is less concerning ~~than initially anticipated~~. However, considering the efforts targeted controlling schistosomiasis over the last decade, the high prevalence of *S. mansoni* alone was surprising and raises significant public health concerns, underscoring the need for enhanced chemotherapy and improved water, sanitation, and hygiene (WASH) services. Reports of synergistic and antagonistic immunological interactions between *S.*

mansoni and *H. pylori* [16,41] prompt further questions about whether these pathogens might exhibit antagonistic relationships at the ecological or behavioral levels, potentially influencing transmission dynamics. This could explain the lower-than-expected co-infection prevalence observed in this study. While our findings are not robust enough to conclusively address this hypothesis, the question merits further investigation to better understand the interplay between these two pathogens in co-endemic settings. In this study, we have reported the prevalence of co-infection with *H. pylori and S. mansoni* infections among school-aged children in five communities in Northern Nigeria. Our findings ~~have provided some form of evidence that~~ are useful in guiding ~~might be important to guide future directions for~~ more robust research and programmatic actions.

Conclusion

Our study highlights an unexpectedly high prevalence of *S. mansoni*, moderate prevalence of H. *pylori*, and very low coinfection prevalence for both. Our findings on *S. mansoni* is particularly concerning, suggesting potential challenges in control programs, that needs investigation. While the co-infection rate was lower than expected, the findings still underscore the need for integrated interventions, including chemotherapy and improved water, sanitation, and hygiene (WASH) services, to address both infections effectively, especially among older children.

Supporting Information ~~lementary~~ Files

S1 File: Dataset

Declarations

Authors' contribution

MF, BA, and HOM conceptualized the study and prepared the protocol, DH approved the protocol. BA and HOM coordinated field surveys. BD, AA were involved in data collection. MF and OO carried out the molecular analysis. HOM performed statistical analysis, and MF and HOM prepared the first draft of the manuscript. All authors contributed to the development of the final manuscript and approved its submission.

~~Funding~~

~~This work received partial support from the Nigerian Institute of Medical Research Grant for studies on Neglected Tropical Diseases [Grant number 0000134].~~

Conflict of Interest

The authors declare that the research was conducted in the absence of any commercial or financial relationships that could be construed as a potential conflict of interest.

Availability of data and materials

The datasets used and/or analyzed are available as supplementary files

Acknowledgements

We are grateful to the community leaders across the study communities, and all the health workers who facilitated the implementation process.

References

1. World Health Organization (WHO). Ending the neglect to attain the Sustainable Development Goals: a road map for neglected tropical diseases 2021–2030. Geneva: World Health Organization; 2020. Licence: CC BY-NC-SA 3.0 IGO.

2. World Health Organization (WHO). Schistosomiasis. 2021. https://www.who.int/news-room/fact-sheets/detail/schistosomiasis. Accessed 20 September 2021.

3. World Health Organization (WHO). Helminth control in school-age children: a guide for managers of control programmes. 2021. https://www.who.int/neglected_diseases/resources/9789241548267/en/. Accessed 20 September 2021.

4. Midzi N, Mduluza T, Chimbar MJ, Tshuma C, Charimar L, et al. Distribution of schistosomiasis and soil transmitted helminthiasis in Zimbabwe: towards a national plan of action for control and elimination. *PLoS Negl Trop Dis*. 2014;**8**, e3014. pmid:25121489

5. McManus, D.P., Dunne, D.W., Sacko, M. *et al.* Schistosomiasis. *Nat Rev Dis Primers* **4**, 13 (2018). https://doi.org/10.1038/s41572-018-0013-8

6. Centre for Disease Control (CDC). Schistosomiasis. Laboratory identification of parasites of public health concern. https://www.cdc.gov/dpdx/schistosomiasis/index.html. Accessed 20 September 2021.

7. Costain AH, MacDonald AS, Smits HH. Corrigendum: Schistosome Egg Migration: Mechanisms, Pathogenesis and Host Immune Responses. *Front. Immunol*. 2019;**10**: 749. pmid:31031753

8. Jose RL. Acute schistosomiasis mansoni: revisited and reconsidered. Mem Inst Oswaldo Cruz, Rio de Janeiro. 2010;**105**(4):422–435. pmid:20721485

9. Guerrant RL, Leite AM, Pinkerton R, Medeiros PH, Cavalcante PA et al. Biomarkers of Environmental Enteropathy, Inflammation, Stunting, and Impaired Growth in Children in Northeast Brazil. *PLoS ONE*. 2016;**11**(9):e0158772. pmid:27690129; PubMed Central PMCID: PMC5045163.

Field Code Changed

Formatted: Font color: Hyperlink

10. Osakunor DNM, Woolhouse MEJ, Mutapi F. Paediatric schistosomiasis: What we know and what we need to know. *PLoS Negl Trop Dis*. 2018;**12**(2): e0006144. pmid:29420537

11. Montresor, A, Crompton, DWT, Hall A, Bundy DAP, Savioli L. Guidelines for the Evaluation of soil-Transmitted Hel-Minthiasis and Schistosomiasis at Community Level: A guide for Managers of Control Programmes. World Health Organization. 1998. Available online: **https://apps.who.int/iris/handle/10665/63821** (accessed on 15 January 2023).

11. Ibrahim A, Ali YBM, Abdel-Aziz A, El-Badry AA. Helicobacter pylori and enteric parasites co-infection among diarrheic and non-diarrheic Egyptian children: seasonality, estimated risks, and predictive factors. *J Parasit Dis*. 2019;**43**(2):198–208.

12. Spotts H, Walelign S, Tesfaye M, Desta K, Tsegaye A, Taye B. Concurrent infection of intestinal parasites and *Helicobacter pylori* among school-age children in Central Ethiopia. *Parasite Epidemiol Control.* 202;**11**:e00177.

13. Smith S, Fowora M, Pellicano R. Infections with *Helicobacter pylori* and challenges encountered in Africa. *World J Gastroenterol*. 2019;**25**(25):3183-3195. doi: 10.3748/wjg.v25.i25.3183. PMID: 31333310; PMCID: PMC6626727.

14. Pellicano R, Ménard A, Rizzetto M, Mégraud F. Helicobacter species and liver diseases: Association or causation? *Lancet Infect Dis*. 2008;**8**:254–260. doi: 10.1016/S1473-3099(08)70066-5.

15. de Korwin JD, Ianiro G, Gibiino G, Gasbarrini A. Helicobacter pylori infection and extragastric diseases in 2017. Helicobacter. 2017:22 Suppl 1. doi: 10.1111/hel.12411

16. Bhattacharjee S, Mejías-Luque R, Loffredo-Verde E, Toska A, Flossdorf M, Gerhard M, Prazeres da Costa C. Concomitant Infection of S. mansoni and H. pylori Promotes Promiscuity of Antigen-Experienced Cells and Primes the Liver for a Lower Fibrotic Response. *Cell Rep*. 2019;**28**(1):231-244.e5. doi: 10.1016/j.celrep.2019.05.108. PMID: 31269443.

17. Ifeanyi O, Adogu P. Prevalence of Helicobacter pylori and other intestinal parasites amongst duodenal and gastric ulcer patients at Imo state University Teaching Hospital, Orlu, south eastern Nigeria. *J Med Med Sci.* 2013;**4**:362–9.

18. Aniekwe O, Jolaiya T, Ajayi A, Adeleye IA, Gerhard M, Smith SI. Co-infection of Helicobacter pylori and intestinal parasites in children of selected low-income

Formatted: Font: (Default) Times New Roman, 12 pt

Formatted: Font: (Default) Times New Roman, 12 pt

Formatted: Font: (Default) Times New Roman, 12 pt

communities in Lagos State, Nigeria. *Parasitol Int.* 2024;**101**:102896. doi: 10.1016/j.parint.2024.102896.

19. Adewale B, Mafe MA, Mogaji HO, Balogun JB, Sulyman MA, Ajayi MB, Akande DO, Balogun EO. Urinary schistosomiasis and anemia among school-aged children from southwestern Nigeria. *Pathog Glob Health*. 2024;**118**(4):325-333. doi: 10.1080/20477724.2024.2322800. Epub 2024 Feb 28. PMID: 38419138; PMCID: PMC11234908.

20. Germani Y, Dauga C, Duval P, Huerre M, Levy M, Pialoux G, Sansonetti P, Grimont PA. Strategy for the detection of Helicobacter species by amplification of 16S rRNA genes and identification of H. felis in a human gastric biopsy. *Research in Microbiology*. 1997;**148**(4):315-26. doi: 10.1016/S0923-2508(97)81587-2.

21. Weiss J, Mecca J, Silva E, Gassner D. Comparison of PCR and other diagnostic techniques for detection of Helicobacter pylori infection in dyspeptic patients. *Journal of Clinical Microbiology.* 1994;**32**:1663–1668. doi: 10.1128/jcm.32.7.1663-1668.1994.

22. Wichmann D, Panning M, Quack T, Kramme S, Burchard GD, Grevelding C, Drosten C. Diagnosing schistosomiasis by detection of cell-free parasite DNA in human plasma. *PLoS Negl Trop Dis*. 2009;**3**(4):e422. doi:10.1371/journal.pntd.0000422

23. Hamburger J, Turetski T, Kapeller I, Deresiewicz R. Highly repeated short DNA sequences in the genome of Schistosoma mansoni recognized by a species-specific probe. *Mol Biochem Parasitol.* 1991;**44**(1):73-80. doi:10.1016/0166-6851(91)90222-r

24. Ikefuna A, Emerenini F, Nwolisa E, Iregbu F, Eke C. Prevalence and risk factors for *Helicobacter pylori* infection among children in Owerri, Nigeria. *Nigerian Journal of Clinical Practice*. 2021;24(8):1188-1193. doi:10.4103/njcp.njcp_687_20

25. Okoroiwu GIA, Okoroiwu IL, Ubosi NI, Sani NM. Sero-prevalence of and risk factors associated with *Helicobacter pylori* infections among individuals with peptic ulcer in Owerri, Imo State, Nigeria during 2020-2021. *African Journal of Clinical and Experimental Microbiology*. 2022;**23**(3):238-247. doi:10.4314/ajcem.v23i3.3

26. Ewa A, Abdulkadir Y, Ibrahim Y. Prevalence of Helicobacter Pylori Among Patients with Chronic Gastritis in Nigeria. *Clinical Medicine Research*. 2024;**13**(6):77-82. doi:10.11648/j.cmr.20241306.12

27. Smith S, Jolaiya T, Fowora M, Palamides P, Ngoka F, Bamidele M, Lesi O, Onyekwere C, Ugiagbe R, Agbo I, Ndububa D, Adekanle O, Adedeji A, Adeleye I, Harrison U. Clinical and Socio- Demographic Risk Factors for Acquisition of *Helicobacter pylori* Infection in Nigeria. *Asian Pacific Journal of Cancer Prevention*. 2018;**19**(7):1851-1857. doi: 10.22034/APJCP.2018.19.7.1851.

28. Holcombe C, Tsimiri S, Eldridge J, Jones DM. Prevalence of antibody to Helicobacter pylori in children in northern Nigeria. *Transactions of the Royal Society of Tropical Medicine and Hygiene*. 1993;**87**(1):19-21. doi:10.1016/0035-9203(93)90403-d

29. Smith SI, Ajayi A, Jolaiya TF, Essiet U. Prevalence, diagnosis and treatment of Helicobacter pylori infection in Nigeria. *Nigerian Journal of Gastroenterology and Hepatology*. 2022;**14**(1):2-10. doi:10.4103/njgh.njgh_10_22

30. Erturk E, Karaman U, Arici Y, Top S, Yolalan G. Factors Influencing Helicobacter Pylori Positivity in Children. Nigerian Journal of Clinical Practice. 2021;**24**(5):685-691. doi:10.4103/njcp.njcp_595_19

31. Chukwuma, O. M., Chukwuma, G. O., Manafa, P. O., Akulue, J. C., & Jeremiah, Z. A. Prevalence and possible risk factors for Helicobacter pylori seropositivity among peptic ulcerative individuals in Nnewi Nigeria. *BioMed Research Journal*. 2020; ***4***(1), 166-172.

32. Hooi JKY, Lai WY, Ng WK, et al. Global Prevalence of Helicobacter pylori Infection: Systematic Review and Meta-Analysis. *Gastroenterology*. 2017;**153**(2):420-429. doi:10.1053/j.gastro.2017.04.022

33. Singh K, Muddasiru D, Singh J. Current status of schistosomiasis in Sokoto, Nigeria. *Parasite Epidemiology and Control*. 2016;**1**(3):239-244. doi:10.1016/j.parepi.2016.08.003

34. Dawaki S, Al-Mekhlafi HM, Ithoi I, et al. The Menace of Schistosomiasis in Nigeria: Knowledge, Attitude, and Practices Regarding Schistosomiasis among Rural Communities in Kano State. *PLoS ONE*. 2015;**10**(11):e0143667. doi:10.1371/journal.pone.0143667

35. Adeniran AA, Mogaji HO, Aladesida AA, et al. Schistosomiasis, intestinal helminthiasis and nutritional status among preschool-aged children in sub-urban communities of Abeokuta, Southwest, Nigeria. *BMC Res Notes*. 2017;**10**(1). doi:10.1186/s13104-017-2973-2

36. Ojo JA, Adedokun SA, Akindele AA, et al. Prevalence of urogenital and intestinal schistosomiasis among school children in South-west Nigeria. *PLoS Negl Trop Dis*. 2021;**15**(7):e0009628. doi:10.1371/journal.pntd.0009628

37. Okeke OC, Akinwale OP, Ubachukwu PO, et al. Report of high prevalence of schistosome infection in Biomphalaria snails from a geographic area with no previous prevalence of human schistosomiasis in Nigeria. *Acta Tropica*. 2019;**210**:105326. doi:10.1016/j.actatropica.2019.105326

38. Ekpo UF, Oluwole AS, Abe EM, Etta HE, Olamiju F, Mafiana CF. Schistosomiasis in infants and pre-school-aged children in sub-Saharan Africa: implication for control. *Parasitology*. 2012;**139**(7):835-841. doi:10.1017/s0031182012000029

39. Eastham G, Fausnacht D, Becker MH, Gillen A, Moore W. Praziquantel resistance in schistosomes: a brief report. *Front Parasitol.* 2024;**3**:1471451. doi: 10.3389/fpara.2024.1471451. PMID: 39817170; PMCID: PMC11732111.

40. Olamiju F, Nebe OJ, Mogaji H, Marcus A, Amodu-Agbi P, Urude RO, Apake E, Olamiju O, Okoronkwo C, Achu I, Mpama O. Schistosomiasis outbreak during COVID-19 pandemic in Takum, Northeast Nigeria: Analysis of infection status and associated risk factors. *PLoS One*. 2022;**17**(1):e0262524. doi: 10.1371/journal.pone.0262524. PMID: 35061823; PMCID: PMC8782311.

41. Elshal MF, Elsayed IH, Kady IME, et al. Role of concurrent S. mansoni infection in H. pylori-associated gastritis: a flow cytometric DNA-analysis and oxyradicals correlations. *Clinica Chimica Acta*. 2004;**346**(2):191-198. doi:10.1016/j.cccn.2004.03.023

RESPONSE TO REVIEWERS COMMENTS

Journal Requirements:

When submitting your revision, we need you to address these additional requirements.

1. Please ensure that your manuscript meets PLOS ONE's style requirements, including those for file naming. The PLOS ONE style templates can be found at https://journals.plos.org/plosone/s/file?id=wjVg/PLOSOne_formatting_sample_main_body.pdf and https://journals.plos.org/plosone/s/file?id=ba62/PLOSOne_formatting_sample_title_authors_affiliations.pdf

Response: We have further revised our text, to ensure they meet the journal guidelines.

2. Please include a complete copy of PLOS' questionnaire on inclusivity in global research in your revised manuscript. Our policy for research in this area aims to improve transparency in the reporting of research performed outside of researchers' own country or community. The policy applies to researchers who have travelled to a different country to conduct research, research with Indigenous populations or their lands, and research on cultural artefacts. The questionnaire can also be requested at the journal's discretion for any other submissions, even if these conditions are not met. Please find more information on the policy and a link to download a blank copy of the questionnaire here: https://journals.plos.org/plosone/s/best-practices-in-research-reporting. Please upload a completed version of your questionnaire as Supporting Information when you resubmit your manuscript.

Response: We have now uploaded a copy of the questionnaire

3. Thank you for stating the following financial disclosure:
This work received partial support from the Nigerian Institute of Medical Research Grant for studies on Neglected Tropical Diseases [Grant number 0000134]. Please state what role the funders took in the study. If the funders had no role, please state: "The funders had no role in study design, data collection and analysis, decision to publish, or preparation of the manuscript." If this statement is not correct you must amend it as needed.

Response: Thanks for this observation. We have now stated that " The funders had no role in study design, data collection and analysis, decision to publish, or preparation of the manuscript."

4. Please include this amended Role of Funder statement in your cover letter; we will change the online submission form on your behalf.

Response; Thanks for this observation. We have now stated that " The funders had no role in study design, data collection and analysis, decision to publish, or preparation of the manuscript." In our cover letter

4. Please note that funding information should not appear in any section or other areas of your manuscript. We will only publish funding information present in the Funding Statement section of the online submission form. Please remove any funding-related text from the manuscript.

Response; We have now removed the funding information from the manuscript.

5. Please amend your authorship list in your manuscript file to include author Hammed Oladeji Mogaji.

Response; The name in the manuscript file is the correct one. We have now corrected the manuscript submission data to reflect **Hammed Mogaji**

6. Please amend the manuscript submission data (via Edit Submission) to include author Hammed Mogaji.

Response; We have now corrected the manuscript submission data to reflect **Hammed Mogaji**

7. Please remove all personal information, ensure that the data shared are in accordance with participant consent, and re-upload a fully anonymized data set.
Note: spreadsheet columns with personal information must be removed and not hidden as all hidden columns will appear in the published file.
Additional guidance on preparing raw data for publication can be found in our Data Policy (https://journals.plos.org/plosone/s/data-availability#loc-human-research-participant-data-and-other-sensitive-data) and in the following article: http://www.bmj.com/content/340/bmj.c181.long.

Response; We have re-uploaded our dataset, and have removed all personal information, or personal identifier. Thanks

8. Please include captions for your Supporting Information files at the end of your manuscript, and update any in-text citations to match accordingly. Please see our Supporting Information guidelines for more information: http://journals.plos.org/plosone/s/supporting-information.

Response; We have included the captions as required.

9. If the reviewer comments include a recommendation to cite specific previously published works, please review and evaluate these publications to determine whether they are relevant and should be cited. There is no requirement to cite these works unless the editor has indicated otherwise.

Response; None of the reviewer requested than we cite any article

REVIEWER 1

The manuscript looks good. The study of the coinfections of Schistosoma mansoni and Helicobacter pylori is a good one and worthy of investigation as the two organisms cohabit gastrointestinal track. This has provided useful information and better understanding on how they impact diseases etiology and this has great public health value and policy implications. The authors should clarify and reconcile the data presented in the manuscript. They should address all comments made on the manuscript.

Response; We are grateful for the very constructive and positive comments of the reviewer. We have now revised the manuscript taking into considerations all the reviewers comments and opinions

Comment 1: Full title is preferable.

Response: The title has been revised

Comment 2: It is not only MDA of praziquantel tablets that are being used for the elimination programme, rather other cross cutting approaches including Integrated Vector Management, WASH, Behavioural Change Communication are all part of the interventions that will facilitate elimination process.

Response: The abstract has also been revised accordingly. The new introductory lines now reads "*Helicobacter pylori* and *Schistosoma mansoni* are two important pathogens that infect the gastrointestinal tract, could be acquired earlier in childhood, and can remain asymptomatic for a long period. While *S. mansoni* is been controlled within routine primary healthcare program, there are no control guidelines for *H. pylori.* Here, we investigate the co-infection pattern of both pathogens to support improvement of management strategies.."

Comment 4: Does this refer to Niger basin or Niger State setting? kindly clarify.

Response: Yes, we have now revised the text to read "Niger State"

Comment 5: The findings for S. mansoni is quite disturbing as several rounds of MDAs have been implemented in Niger State. This calls to question the quality of intervention implemented all these years. The authors should have investigated the MDA history, therapeutic and geographical reach

Response: Yes, we agree absolutely with the reviewer, and noted this in our discussion. This observation is worth further investigation, and public health action.

Comments: Line 127-128: Why variation in the sample size

Response: We thank the reviewer for this important observation. This study is a sub-study, that follows a larger study where 410 children were recruited. However, for this sub-study only 73% consented to participate in this study. i.e. 100% compliance across all the communities except Monai (16.5% compliance). We have revised the sample-size estimation sub-section to provide more clarity.

Comments: Line 180-181: Kindly reconcile this number 299 with the earlier number of participants 410 under the smaple size

Response: We thank the reviewer for this important observation. This study is a sub-study, that follows a larger study where 410 children were recruited. However, for this sub-study only 73% consented to participate in this study. i.e. 100% compliance across all the communities except Monai (16.5% compliance). We have revised the sample-size estimation sub-section to provide more clarity.

Comments: Table 1, See comment above

Response: We thank the reviewer for this important observation. We have provided the response to this comment in our previous response.

Comments; Line 247-249: Kindly reconcile this statistics with the FMOH data base on schistosomiasis prevalence 2013-2017mappimg data. It is available with the National Programme and WHO ESPEN.

Response: We thank the reviewer for this important observation. We have revised the text to emphasize our comparison with research findings..

REVIEWER 2:

C1: Study overview

This study attempts to evaluate co-infections of Schistosoma mansoni and Helicobacter pylori among school-aged populations across five communities. Although, I find this initiative timely and important, the manuscript does not satisfactorily pitch the link between the two pathogens in the introduction, despite reporting findings that could possibly inform future interventions. I hope my somewhat extensive comments do not discourage the authors and that they are received in the constructive and supportive spirit in which they are offered. For clarity, I have divided my comments into three categories, which I hope should help improve the overall presentation of your manuscript.

RESPONSE: We thank the reviewer for the comments, which have significantly improved the quality of our manuscript. We have carefully incorporated all the review suggestions made and hope you find the manuscript worthy of publication.

General comments:

1. It is not clear why these two pathogens are studied together. The manuscript does not precisely describe whether they share transmission pathway, immunological interaction etc. I expected to see a clearer rationale linking the topic and objective.

Response: We thank the reviewer for this comment. In our manuscript between lines 74-80, we describe the transmission pathway, clinical manifestations and speculations of altered disease pathology. We cited 6 references within this space. Further between lines 82-91, we further linked these lines with the rationale of generating evidence that improves the management of both diseases.

2. Also, I expected to read key findings that could inform changes in the management of both pathogens.

Response: We thank the reviewers for this comment. Following our analysis, we reported an unexpectedly high prevalence of *S. mansoni*, moderate prevalence of H. *pylori*, and very low coinfection prevalence for both. Our findings on *S. mansoni* is particularly concerning, suggesting potential challenges in control programs that needs investigation. While the co-infection rate was lower than expected, the findings still underscore the need for integrated interventions, including chemotherapy and improved water, sanitation, and hygiene (WASH) services, to address both infections effectively, especially among older children. These excerpts are currently in the abstract (lines; 32-43; results; discussion; conclusion (lines 271-277)

Comments 3. In the Methods section, the collection of data on water contact activities

and parent's occupation was mentioned. However, these variables were neither included in the analysis nor reported in the Results and Discussion. Their omission, along with the absence of other important variables such as proxies for overcrowding and WASH indicators, somewhat weakens the manuscript, as such factors are crucial for guiding interventions.

Response: We appreciate the reviewer for this observation and clearly acknowledge this gap. We have revised these lines by deleting the variables that were not collected. Also, we added the context of the five study communities between lines 113-115 based on the previous study conducted amongst this population. The poor WASH access in the population still justifies the recommendations given, since our participants were drawn out (i.e., subsets) of that locality. Please see lines 124-126, and lines 133-137.

Comment 4. To emphasize the importance of this finding, consider noting in the Discussion (perhaps in the last paragraph) that schistosomiasis is classified as a Neglected Tropical Disease (NTD) according to the World Health Organization (WHO). This will highlight the importance of your work in identifying the locations and age groups with the highest prevalence odds of infection, thereby providing evidence to guide targeted interventions.

Response: We thank the reviewer for this comment. We have added a line to the last paragraph (please see line 264), and also introduced schistosomiasis as a NTD in the first line of our introduction.

Major comments

Comment 1. The introduction reads a bit underdeveloped. The literature review should be expanded to situate the study within existing knowledge. For example, lines 74-76 should more robustly explain the “similar risk” described. While both pathogens are WASH-related GIT, H. pylori is chiefly fecal-oral, oral-oral route often in childhood, while S. mansoni is via skin penetration by cercariae in contaminated water – both possessing different risk factors. As such, significant improvement is need in the introduction to satisfactorily present the study especially given what the study intend to present.

Response: We appreciate the reviewer for this amazing comment, which was super useful. We have revised that paragraph to illustrate both the commonality and differences in their risk factors. Please see Line 75-79

Comments 2. Lines 234-236: Given that H. pylori infection is commonly reported during childhood and increases with age, the 1% seropositivity reported in Monai may be better explained by other unreported variables. Since these were omitted, the title should perhaps be revised to reflect the actual scope (demographic-related), rather than implying analyses that were not performed.

Response: We thank the reviewer for this comment. Considering the revisions we made, We have now provided the overall occupation, access to latrine and walking barefoot for these 5 communities studies. Based on this access to sanitation in Monai was 22%, and this resonates with the lines we have on Monai.

Comments 3. Consider starting your Discussion section with the presentation of your “principal findings”, as this would enhance the quality of your manuscript. This way, you could move the first two sentences under Discussion to the latter part of the manuscript.

Response: We thank the reviewer for this comment. We have now moved the two sentences to the latter part of the manuscript

Comments 4. Lines 240 and 254: The text suggests a higher risk of co-infection among older children, which is inconsistent with both the study design and findings. The measure used in this study is OR, which captures “odds”, and not “risk”. Also, "Older children" is vague and not previously defined. The statement also overlooks the fact that no statistically significant evidence was found for the 15-17 age group. I recommend replacing "risk" with "chance". If the term "younger children" and "older children" are to be retained, age ranges should be defined in the Methods section. Otherwise, refer to the age ranges in the Discussion as they appear in the tables.

Response: We thank the reviewer for this comment. This is such an amazing observation. We have revised the text completely to replace risk with chance, and also to replace the younger or older children with the exact age categories as they appear in the tables. Please see Lines 240-266.

Minor comments

Comments 1. Keywords: It would be advisable to select keywords that do not overlap with the words in the title of the article, to improve discoverability during search.

Response: We thank the reviewer for these comments. This is well noted

Comments 2. Lines 24- 26: The statement appears awkward and incomplete, lacking logical flow between the two pathogens. The authors should attempt to establish explicit mechanistic link explaining why these pathogens are being co-investigated.

Response: We thank the reviewer for these comments. We have now revised the abstract to portray clarity.

Comments 3. Consider moving the ethical state to the final part of Methods, as is the common practice

Response: We thank the reviewer for these comments. We have now moved it.

Comments 4. The phrase “Niger area” is ambiguous, are you referring to Niger State, the River Niger basin, or another region? Please clarify.

Response: We thank the reviewer for these comments. We have now corrected this. We meant Niger State.

Comments: 5. Please justify the use of real-time PCR for S. mansoni and conventional PCR for H. pylori, especially since PCR detection of H. pylori is less common than antigen or urea breath testing.

Response: We thank the reviewer for these comments. We agree that using H. pylori detection is less common than antigen or urea breath testing. However, we already had primers and other reagents for the molecular detection in our laboratory. In the face of limited funding for this work, we resulted to using PCR detection that was readily available in our laboratory. With regards to the use of qPCR for S. mansoni detection, studies have shown that RT-PCR was the most sensitive diagnostic method and gave the highest positive detection of Schistosoma species in stool specimen (Guegan et al. 2019). Hence, the use of RT-PCR for the detection of Schistosoma manosnii in this study.

Reference

Guegan H, Fillaux J, Charpentier E, Robert-Gangneux F, Chauvin P, Guemas E, et al. (2019) Real-time PCR for diagnosis of imported schistosomiasis. PLoS Negl Trop Dis 13(9): e0007711. https://doi.org/10.1371/journal.pntd.0007711

Comments; 6. The abstract overly descriptive. To enrich it, kindly incorporate a clear discussion of the key findings and potential explanations.

Response: We thank the reviewer for these comments. We have now revised the abstract to include these key findings as requested

Comments 7. Lines 101-103: Please specify what type of consent obtained and the database repository used.

Response: We thank the reviewer for this comment. Informed consent was obtained from parents/guardian and children. Children's assent was obtained verbally and documented via thumb print through an assent form in the presence of the parent or legal guardian who provided written consent. We used a password protected computer to create and assign unique ID

Comments 8. Lines 112 – 114: Please provide a reference to support this claim.

Response: We thank the reviewer for these comments. We have now provided the clear metrics and also he reference for this

Comments 9. There is a slight inconsistency in reported age of participants. E.g. the Methods states 6-16 yrs, but the Results report 6-17 yrs. Please address this discrepancy.

Response: We thank the reviewer for these comments. We have now revised the text to 6-17 years. key findings as requested

Comments: 10. The sample size justification is vague, lacking essential parameters such as expected prevalence and margin of error.

Response: We thank the reviewer for these comments. The sample size estimation followed WHO guidelines of recruiting 50 pupils per school/villages. And we adjusted this size by 60% to cater for non-enrolled school aged children. However, of the 410 children recruited for the larger project, only 73% consented to participate in this study. i.e. 100% compliance across all the communities except Monai (16.5% compliance).

Comments: 11. Line 140: Were participants instructed to provide stool samples at a specific time of the day, or was any sample acceptable? Given the collection period, how was sample quality and integrity ensured? Why was repeated sampling not considered, since egg shedding S. mansoni can be intermittent?

Response; Thanks for the comments. Yes, only fresh samples were retrieved, as detailed between lines 164-166. Samples were collected within a very limited time frame of 1hr, and the parents/legal guardians supervised the voiding process. The need for sample integrity was sufficiently explained to the parents and their children.

Comments 12. Line 142: Define “NIMR” at the first mention.

Response; Thanks for the comments. This has now been addressed

13. Kindly ensure that all scientific names are properly italicized following general taxonomy rules. There are sporadic occasions where this was not done in the manuscript (e.g. lines 147 and 148). Also, remove italics from “and” in “H. pylori and S. mansoni infections” at lines 193 and 205.

Response; Thanks for the comments. This has now been addressed

Comments 14. Kindly consider adding a minimal description of the variables included in the models is missing. What covariates were used? The supplementary materials do not include a data dictionary explaining each variable, and this information should still be included in the Methods since readers may not fully understand what is being tested.

Response; Thanks for the comments. This has now been addressed

Comments 15. Generally, reporting both p-values and confidence intervals together is somewhat redundant (e.g., lines 174 and 175, as well as throughout the Results section). If opting to continue with p-values, kindly standardize it throughout the manuscript, presenting all to three 3 significant digits.

Response; Thanks for the comments. We have standardized the p-value to 3 digits cross the text. However, we prefer to retain both the CI, to demonstrate precision around the estimate, and the p-value to demonstrate significance.

Comments 16. Line 183: The phrasing suggests a decreasing sequence, but this is not the case.

Response; Thanks for the comments. We have now addressed it

17. Add footnotes to tables defining all terms and abbreviations.

Response; Thanks for the comments. We have now addressed it

18. Line 220: It would be more appropriate and academically formal to use “Odds Ratio”.

Response; Thanks for the comments. We have now addressed it

19. Lines 224-225: Briefly specify what the “some evidence that might be important [...]” are

Response; Thanks for the comments. We have now addressed it. We moved this to the last paragraph as you earlier suggested and also, we rephrased it s’ our findings are useful in guiding more robust research and programmatic actions.

20. Line 227: “similar those”, please revise.

Response; Thanks for the comments. We have now addressed it

21. Lines 230-232: The justification here is unconvincing. Niger State is also in Northern Nigeria, and you have not conducted or presented any socio-environmental analysis to substantiate this claim.

Response; Thanks for the comments. Our comments here refer to the differences between our study and several other study in Nigeria that we cited. We were only trying to suggest the differences might be related to the diagnostic tool (as indeed we used PCR, which are more sensitive), and hygiene standard of the participants or sites.

22. While the use of ORs in cross-sectional studies is common, this being a prevalence study, results may risk overestimation. In this case, Prevalence Ratios (PRs) might provide a more accurate measure of association. However, I do not discourage the use of ORs, but it would be worthwhile to mention this as a limitation.

Response; Thanks for the comments., and we appreciate the reviewers concern on this.

23. Line 260: What exactly was “previously predicted”? No hypotheses were explicitly stated earlier.

Response; Thanks for the comments. We have now addressed it by removing that

24. Per journal policy, please deposit the script and supporting data in an open repository.

Response; Thanks for the comments. We have provided the supporting data.
