# Supplementary material for: Co-infections of Schistosoma mansoni and Helicobacter pylori in school-aged populations and implication for management and control practices in Niger State, Nigeria
Source: PLoS One. 2025 Dec 8;20(12):e0337423. doi: 10.1371/journal.pone.0337423 (PMC12685161; doi:10.1371/journal.pone.0337423)
Supplement: Attachment — Submitted filename: Response to Reviewer.docx [file pone.0337423.s007.docx]

**RESPONSE TO REVIEWERS COMMENTS**

**Journal Requirements:**

When submitting your revision, we need you to address these additional requirements.

1. Please ensure that your manuscript meets PLOS ONE's style requirements, including those for file naming. The PLOS ONE style templates can be found at <https://journals.plos.org/plosone/s/file?id=wjVg/PLOSOne_formatting_sample_main_body.pdf> and <https://journals.plos.org/plosone/s/file?id=ba62/PLOSOne_formatting_sample_title_authors_affiliations.pdf>

**Response**: We have further revised our text, to ensure they meet the journal guidelines.

2. Please include a complete copy of PLOS’ questionnaire on inclusivity in global research in your revised manuscript. Our policy for research in this area aims to improve transparency in the reporting of research performed outside of researchers’ own country or community. The policy applies to researchers who have travelled to a different country to conduct research, research with Indigenous populations or their lands, and research on cultural artefacts. The questionnaire can also be requested at the journal’s discretion for any other submissions, even if these conditions are not met.  Please find more information on the policy and a link to download a blank copy of the questionnaire here: <https://journals.plos.org/plosone/s/best-practices-in-research-reporting>. Please upload a completed version of your questionnaire as Supporting Information when you resubmit your manuscript.

**Response:** We have now uploaded a copy of the questionnaire

3. Thank you for stating the following financial disclosure:
This work received partial support from the Nigerian Institute of Medical Research Grant for studies on Neglected Tropical Diseases [Grant number 0000134]. Please state what role the funders took in the study. If the funders had no role, please state: "The funders had no role in study design, data collection and analysis, decision to publish, or preparation of the manuscript." If this statement is not correct you must amend it as needed.

**Response: Thanks for this observation. We have now stated that “** The funders had no role in study design, data collection and analysis, decision to publish, or preparation of the manuscript."

4. Please include this amended Role of Funder statement in your cover letter; we will change the online submission form on your behalf.

**Response; Thanks for this observation. We have now stated that “** The funders had no role in study design, data collection and analysis, decision to publish, or preparation of the manuscript." In our cover letter

4. Please note that funding information should not appear in any section or other areas of your manuscript. We will only publish funding information present in the Funding Statement section of the online submission form. Please remove any funding-related text from the manuscript.

**Response;** We have now removed the funding information from the manuscript.

 5. Please amend your authorship list in your manuscript file to include author Hammed Oladeji Mogaji.

**Response;** The name in the manuscript file is the correct one. We have now corrected the manuscript submission data to reflect **Hammed Mogaji**

6. Please amend the manuscript submission data (via Edit Submission) to include author Hammed Mogaji.

**Response;** We have now corrected the manuscript submission data to reflect **Hammed Mogaji**

 7. [Please remove all personal information, ensure that the data shared are in accordance with participant consent, and re-upload a fully anonymized data set.
 Note: spreadsheet columns with personal information must be removed and not hidden as all hidden columns will appear in the published file.
Additional guidance on preparing raw data for publication can be found in our Data Policy (https://journals.plos.org/plosone/s/data-availability#loc-human-research-participant-data-and-other-sensitive-data) and in the following article: http://www.bmj.com/content/340/bmj.c181.long.](Please%20remove%20all%20personal%20information,%20ensure%20that%20the%20data%20shared%20are%20in%20accordance%20with%20participant%20consent,%20and%20re-upload%20a%20fully%20anonymized%20data%20set.%20Note:%20spreadsheet%20columns%20with%20personal%20information%20must%20be%20removed%20and%20not%20hidden%20as%20all%20hidden%20columns%20will%20appear%20in%20the%20published%20file.Additional%20guidance%20on%20preparing%20raw%20data%20for%20publication%20can%20be%20found%20in%20our%20Data%20Policy%20(https://journals.plos.org/plosone/s/data-availability#loc-human-research-participant-data-and-other-sensitive-data) and in the following article: http://www.bmj.com/content/340/bmj.c181.long.)

**Response;** We have re-uploaded our dataset, and have removed all personal information, or personal identifier. Thanks

8. Please include captions for your Supporting Information files at the end of your manuscript, and update any in-text citations to match accordingly. Please see our Supporting Information guidelines for more information: http://journals.plos.org/plosone/s/supporting-information.

**Response**; We have included the captions as required.

9. If the reviewer comments include a recommendation to cite specific previously published works, please review and evaluate these publications to determine whether they are relevant and should be cited. There is no requirement to cite these works unless the editor has indicated otherwise.

**Response;** None of the reviewer requested than we cite any article

**REVIEWER 1**

The manuscript looks good. The study of the coinfections of Schistosoma mansoni and Helicobacter pylori is a good one and worthy of investigation as the two organisms cohabit gastrointestinal track. This has provided useful information and better understanding on how they impact diseases etiology and this has great public health value and policy implications. The authors should clarify and reconcile the data presented in the manuscript. They should address all comments made on the manuscript.

**Response;** We are grateful for the very constructive and positive comments of the reviewer. We have now revised the manuscript taking into considerations all the reviewers comments and opinions

Comment 1: Full title is preferable.

**Response**: The title has been revised

Comment 2: It is not only MDA of praziquantel tablets that are being used for the elimination programme, rather other cross cutting approaches including Integrated Vector Management, WASH, Behavioural Change Communication are all part of the interventions that will facilitate elimination process.

**Response**: The abstract has also been revised accordingly. The new introductory lines now reads “*Helicobacter pylori* and *Schistosoma mansoni* are two important pathogens that infect the gastrointestinal tract, could be acquired earlier in childhood, and can remain asymptomatic for a long period. While *S. mansoni* is been controlled within routine primary healthcare program, there are no control guidelines for *H. pylori.* Here, we investigate the co-infection pattern of both pathogens to support improvement of management strategies..”

Comment 4: Does this refer to Niger basin or Niger State setting? kindly clarify.

**Response:** Yes, we have now revised the text to read “Niger State”

Comment 5: The findings for S. mansoni is quite disturbing as several rounds of MDAs have been implemented in Niger State. This calls to question the quality of intervention implemented all these years. The authors should have investigated the MDA history, therapeutic and geographical reach

**Response**: Yes, we agree absolutely with the reviewer, and noted this in our discussion. This observation is worth further investigation, and public health action.

Comments: Line 127-128: Why variation in the sample size

**Response:** We thank the reviewer for this important observation. This study is a sub-study, that follows a larger study where 410 children were recruited. However, for this sub-study only 73% consented to participate in this study. i.e. 100% compliance across all the communities except Monai (16.5% compliance). We have revised the sample-size estimation sub-section to provide more clarity.

Comments: Line 180-181: Kindly reconcile this number 299 with the earlier number of participants 410 under the smaple size

**Response:** We thank the reviewer for this important observation. This study is a sub-study, that follows a larger study where 410 children were recruited. However, for this sub-study only 73% consented to participate in this study. i.e. 100% compliance across all the communities except Monai (16.5% compliance). We have revised the sample-size estimation sub-section to provide more clarity.

Comments: Table 1, See comment above

**Response:** We thank the reviewer for this important observation. We have provided the response to this comment in our previous response.

Comments; Line 247-249: Kindly reconcile this statistics with the FMOH data base on schistosomiasis prevalence 2013-2017mappimg data. It is available with the National Programme and WHO ESPEN.

**Response:** We thank the reviewer for this important observation. We have revised the text to emphasize our comparison with research findings..

**REVIEWER 2:**

C1:  Study overview
This study attempts to evaluate co-infections of Schistosoma mansoni and Helicobacter pylori among school-aged populations across five communities. Although, I find this initiative timely and important, the manuscript does not satisfactorily pitch the link between the two pathogens in the introduction, despite reporting findings that could possibly inform future interventions. I hope my somewhat extensive comments do not discourage the authors and that they are received in the constructive and supportive spirit in which they are offered. For clarity, I have divided my comments into three categories, which I hope should help improve the overall presentation of your manuscript.

**RESPONSE**: We thank the reviewer for the comments, which have significantly improved the quality of our manuscript. We have carefully incorporated all the review suggestions made and hope you find the manuscript worthy of publication.

General comments:
1. It is not clear why these two pathogens are studied together. The manuscript does not precisely describe whether they share transmission pathway, immunological interaction etc. I expected to see a clearer rationale linking the topic and objective.

**Response:** We thank the reviewer for this comment. In our manuscript between lines 74-80, we describe the transmission pathway, clinical manifestations and speculations of altered disease pathology. We cited 6 references within this space. Further between lines 82-91, we further linked these lines with the rationale of generating evidence that improves the management of both diseases.

2. Also, I expected to read key findings that could inform changes in the management of both pathogens.

**Response:** We thank the reviewers for this comment. Following our analysis, we reported an unexpectedly high prevalence of *S. mansoni*, moderate prevalence of H*. pylori*, and very low coinfection prevalence for both. Our findings on *S. mansoni* is particularly concerning, suggesting potential challenges in control programs that needs investigation. While the co-infection rate was lower than expected, the findings still underscore the need for integrated interventions, including chemotherapy and improved water, sanitation, and hygiene (WASH) services, to address both infections effectively, especially among older children. These excerpts are currently in the abstract (lines; 32-43; results; discussion; conclusion (lines 271-277)

Comments 3. In the Methods section, the collection of data on water contact activities and parent’s occupation was mentioned. However, these variables were neither included in the analysis nor reported in the Results and Discussion. Their omission, along with the absence of other important variables such as proxies for overcrowding and WASH indicators, somewhat weakens the manuscript, as such factors are crucial for guiding interventions.

**Response:** We appreciate the reviewer for this observation and clearly acknowledge this gap. We have revised these lines by deleting the variables that were not collected. Also, we added the context of the five study communities between lines 113-115 based on the previous study conducted amongst this population. The poor WASH access in the population still justifies the recommendations given, since our participants were drawn out (i.e., subsets) of that locality. Please see lines 124-126, and lines 133-137.

Comment 4. To emphasize the importance of this finding, consider noting in the Discussion (perhaps in the last paragraph) that schistosomiasis is classified as a Neglected Tropical Disease (NTD) according to the World Health Organization (WHO). This will highlight the importance of your work in identifying the locations and age groups with the highest prevalence odds of infection, thereby providing evidence to guide targeted interventions.

**Response:** We thank the reviewer for this comment. We have added a line to the last paragraph (please see line 264), and also introduced schistosomiasis as a NTD in the first line of our introduction.

**Major comments**

Comment 1. The introduction reads a bit underdeveloped. The literature review should be expanded to situate the study within existing knowledge. For example, lines 74-76 should more robustly explain the “similar risk” described. While both pathogens are WASH-related GIT, H. pylori is chiefly fecal-oral, oral-oral route often in childhood, while S. mansoni is via skin penetration by cercariae in contaminated water – both possessing different risk factors. As such, significant improvement is need in the introduction to satisfactorily present the study especially given what the study intend to present.

**Response:** We appreciate the reviewer for this amazing comment, which was super useful. We have revised that paragraph to illustrate both the commonality and differences in their risk factors. Please see Line 75-79

Comments 2. Lines 234-236: Given that H. pylori infection is commonly reported during childhood and increases with age, the 1% seropositivity reported in Monai may be better explained by other unreported variables. Since these were omitted, the title should perhaps be revised to reflect the actual scope (demographic-related), rather than implying analyses that were not performed.

**Response:** We thank the reviewer for this comment. Considering the revisions we made, We have now provided the overall occupation, access to latrine and walking barefoot for these 5 communities studies. Based on this access to sanitation in Monai was 22%, and this resonates with the lines we have on Monai.

Comments 3. Consider starting your Discussion section with the presentation of your “principal findings”, as this would enhance the quality of your manuscript. This way, you could move the first two sentences under Discussion to the latter part of the manuscript.

**Response:** We thank the reviewer for this comment. We have now moved the two sentences to the latter part of the manuscript

Comments 4. Lines 240 and 254: The text suggests a higher risk of co-infection among older children, which is inconsistent with both the study design and findings. The measure used in this study is OR, which captures “odds”, and not “risk”. Also, "Older children" is vague and not previously defined. The statement also overlooks the fact that no statistically significant evidence was found for the 15-17 age group. I recommend replacing "risk" with "chance". If the term "younger children" and "older children" are to be retained, age ranges should be defined in the Methods section. Otherwise, refer to the age ranges in the Discussion as they appear in the tables.

**Response:** We thank the reviewer for this comment. This is such an amazing observation. We have revised the text completely to replace risk with chance, and also to replace the younger or older children with the exact age categories as they appear in the tables. Please see Lines 240-266.

**Minor comments**

Comments 1. Keywords: It would be advisable to select keywords that do not overlap with the words in the title of the article, to improve discoverability during search.

**Response:** We thank the reviewer for these comments. This is well noted

Comments 2. Lines 24- 26: The statement appears awkward and incomplete, lacking logical flow between the two pathogens. The authors should attempt to establish explicit mechanistic link explaining why these pathogens are being co-investigated.

**Response:** We thank the reviewer for these comments. We have now revised the abstract to portray clarity.

Comments 3. Consider moving the ethical state to the final part of Methods, as is the common practice

**Response:** We thank the reviewer for these comments. We have now moved it.

Comments 4. The phrase “Niger area” is ambiguous, are you referring to Niger State, the River Niger basin, or another region? Please clarify.

**Response:** We thank the reviewer for these comments. We have now corrected this. We meant Niger State.

Comments: 5. Please justify the use of real-time PCR for S. mansoni and conventional PCR for H. pylori, especially since PCR detection of H. pylori is less common than antigen or urea breath testing.

**Response:** We thank the reviewer for these comments. We agree that using H. pylori detection is less common than antigen or urea breath testing. However, we already had primers and other reagents for the molecular detection in our laboratory. In the face of limited funding for this work, we resulted to using PCR detection that was readily available in our laboratory. With regards to the use of qPCR for S. mansoni detection, studies have shown that RT-PCR was the most sensitive diagnostic method and gave the highest positive detection of Schistosoma species in stool specimen (Guegan et al. 2019). Hence, the use of RT-PCR for the detection of Schistosoma manosnii in this study.

Reference

Guegan H, Fillaux J, Charpentier E, Robert-Gangneux F, Chauvin P, Guemas E, et al. (2019) Real-time PCR for diagnosis of imported schistosomiasis. PLoS Negl Trop Dis 13(9): e0007711. https://doi.org/10.1371/journal.pntd.0007711

Comments; 6. The abstract overly descriptive. To enrich it, kindly incorporate a clear discussion of the key findings and potential explanations.

**Response:** We thank the reviewer for these comments. We have now revised the abstract to include these key findings as requested

Comments 7. Lines 101-103: Please specify what type of consent obtained and the database repository used.

**Response:** We thank the reviewer for this comment. Informed consent was obtained from parents/guardian and children. Children’s assent was obtained verbally and documented via thumb print through an assent form in the presence of the parent or legal guardian who provided written consent. We used a password protected computer to create and assign unique ID

Comments 8. Lines 112 – 114: Please provide a reference to support this claim.

**Response:** We thank the reviewer for these comments. We have now provided the clear metrics and also he reference for this

Comments 9. There is a slight inconsistency in reported age of participants. E.g. the Methods states 6-16 yrs, but the Results report 6-17 yrs. Please address this discrepancy.

**Response:** We thank the reviewer for these comments. We have now revised the text to 6-17 years. key findings as requested

Comments: 10. The sample size justification is vague, lacking essential parameters such as expected prevalence and margin of error.

**Response:** We thank the reviewer for these comments. The sample size estimation followed WHO guidelines of recruiting 50 pupils per school/villages. And we adjusted this size by 60% to cater for non-enrolled school aged children. However, of the 410 children recruited for the larger project, only 73% consented to participate in this study. i.e. 100% compliance across all the communities except Monai (16.5% compliance).

Comments: 11. Line 140: Were participants instructed to provide stool samples at a specific time of the day, or was any sample acceptable? Given the collection period, how was sample quality and integrity ensured? Why was repeated sampling not considered, since egg shedding S. mansoni can be intermittent?

**Response;** Thanks for the comments. Yes, only fresh samples were retrieved, as detailed between lines 164-166. Samples were collected within a very limited time frame of 1hr, and the parents/legal guardians supervised the voiding process. The need for sample integrity was sufficiently explained to the parents and their children.

Comments 12. Line 142: Define “NIMR” at the first mention.

**Response;** Thanks for the comments. This has now been addressed

13. Kindly ensure that all scientific names are properly italicized following general taxonomy rules. There are sporadic occasions where this was not done in the manuscript (e.g. lines 147 and 148). Also, remove italics from “and” in “H. pylori and S. mansoni infections” at lines 193 and 205.

**Response;** Thanks for the comments. This has now been addressed

Comments 14. Kindly consider adding a minimal description of the variables included in the models is missing. What covariates were used? The supplementary materials do not include a data dictionary explaining each variable, and this information should still be included in the Methods since readers may not fully understand what is being tested.

**Response;** Thanks for the comments. This has now been addressed

Comments 15. Generally, reporting both p-values and confidence intervals together is somewhat redundant (e.g., lines 174 and 175, as well as throughout the Results section). If opting to continue with p-values, kindly standardize it throughout the manuscript, presenting all to three 3 significant digits.

**Response;** Thanks for the comments. We have standardized the p-value to 3 digits cross the text. However, we prefer to retain both the CI, to demonstrate precision around the estimate, and the p-value to demonstrate significance.

Comments 16. Line 183: The phrasing suggests a decreasing sequence, but this is not the case.

**Response;** Thanks for the comments. We have now addressed it

17. Add footnotes to tables defining all terms and abbreviations.

**Response;** Thanks for the comments. We have now addressed it

18. Line 220: It would be more appropriate and academically formal to use “Odds Ratio”.

**Response;** Thanks for the comments. We have now addressed it

19. Lines 224-225: Briefly specify what the “some evidence that might be important [...]” are

**Response;** Thanks for the comments. We have now addressed it. We moved this to the last paragraph as you earlier suggested and also, we rephrased it s’ our findings are useful in guiding more robust research and programmatic actions.

20. Line 227: “similar those”, please revise.

**Response;** Thanks for the comments. We have now addressed it

21. Lines 230-232: The justification here is unconvincing. Niger State is also in Northern Nigeria, and you have not conducted or presented any socio-environmental analysis to substantiate this claim.

**Response;** Thanks for the comments. Our comments here refer to the differences between our study and several other study in Nigeria that we cited. We were only trying to suggest the differences might be related to the diagnostic tool (as indeed we used PCR, which are more sensitive), and hygiene standard of the participants or sites.

22. While the use of ORs in cross-sectional studies is common, this being a prevalence study, results may risk overestimation. In this case, Prevalence Ratios (PRs) might provide a more accurate measure of association. However, I do not discourage the use of ORs, but it would be worthwhile to mention this as a limitation.

**Response;** Thanks for the comments., and we appreciate the reviewers concern on this.

23. Line 260: What exactly was “previously predicted”? No hypotheses were explicitly stated earlier.

**Response;** Thanks for the comments. We have now addressed it by removing that

24. Per journal policy, please deposit the script and supporting data in an open repository.

**Response;** Thanks for the comments. We have provided the supporting data.
