# Supplementary material for: Co-infections of Schistosoma mansoni and Helicobacter pylori in school-aged populations and implication for management and control practices in Niger State, Nigeria
Source: PLoS One. 2025 Dec 8;20(12):e0337423. doi: 10.1371/journal.pone.0337423 (PMC12685161; doi:10.1371/journal.pone.0337423)
Supplement: Attachment — Submitted filename: RESPONSE TO REVIEWERS_r2.docx [file pone.0337423.s008.docx]

RESPONSE TO REVIEWERS _ROUND 2

**Reviewer #1:**

There are minor corrections such as scientific names of organisms not italicized in accordance with general taxonomy rules, kindly check all the scientific names in the manuscript and ensure that they are in italics.

Response: We thank the reviewer for this comment, and have carefully revised the manuscript to ensure the scientific names are italicized

**Reviewer #2:**

 Study overview
The authors are commended for thoroughly addressing the comments from the initial round of review. The revision has notably improved the overall quality and presentation of the manuscript, congratulations on this progress.
The central idea of the study remains valuable and of high relevance, however, ONE MAJOR concern persists regarding the lack of key information necessary to adequately test the promised objective to “evaluate the prevalence and associated risk factors of H. pylori and S. mansoni co-infection …”. The study could be further strengthened by including specific additional participant-level information, such as hygiene-related and socio-environmental factors etc, probably from the bigger project. Incorporating these variables would allow for a more robust analysis, richer results and discussion, and ultimately a much stronger manuscript with greater potential to inform policy decisions.

Response: We thank the reviewer for this insightful comment and acknowledge the importance of their suggestion. We agree that incorporating additional covariates could enhance the robustness of the models. However, the current study was primarily designed to describe the co-infection patterns of both pathogens and to assess differences based on age and sex characteristics. We have revised Line 92 to clarify this focus and have also added a section under the study’s limitations to acknowledge this constraint.

Major comments
1.The statistical analysis and the lack of key data necessary to strengthen the manuscript’s objective are somewhat concerning. For example, the analysis appears to rely primarily on limited demographic variables (age and sex only), which are insufficient to fully address the stated objective. Crucial participant-level information that would allow for a more robust evaluation of potential risk factors, as outlined in the objective, is missing.
Similarly, the exclusive use of univariate logistic regression limits the analytical depth of the study. While this approach can identify relationships/associations between individual independent variables and the outcome variable, it does not account for confounding factors that may influence these relationships. As such, conducting a multivariable regression analysis that adjusts for relevant confounders is strongly recommended, as this would greatly enhance the analytical rigor and overall quality of the manuscript.
If additional data cannot be obtained or a multivariable analysis cannot be performed, it is suggested that the authors revise that study objective to clarify that the analysis is purely descriptive and limited to evaluating associations between the two pathogens and basic demographic factors. However, this alternative would somewhat reduce the manuscript’s scientific strength.

Response: We appreciate the reviewer’s comment. As stated in the manuscript, our primary objective was to explore associations based on age and gender rather than to develop a comprehensive predictive model, which would require additional covariates. This approach may be considered in future studies. Additionally, since only two covariates were included and one of them (gender) was not significantly associated (p-value > 0.2), including both in a multivariate model would not meaningfully change the interpretation. Furthermore, with no evidence of potential confounding, adjusting for gender would not alter the effect of age. Therefore, the univariate model presented provides a parsimonious and stable estimate of the association.

2.Consider splitting and refining/improving the second paragraph to (i) dedicate one paragraph specifically to H. pylori similar to how you focused on Schistosoma spp. in the first paragraph, (ii) use the subsequent paragraph to discuss the potential co-infection between both pathogens, which would help enhance the contextual depth and clarity of the manuscript.

**Response:** We thank the reviewer for this comment. Our introduction is currently two pages long, and we aim to keep it within this limit while maintaining a logical flow. The intent was to build a case for improved management of *H. pylori* infections as part of routine schistosomiasis control programs. To achieve this, we began with a paragraph on schistosomiasis and linked it to *H. pylori*. The second paragraph focused primarily on *H. pylori*, while connecting its epidemiology with schistosomiasis. Between lines 77–83, we discussed predilection sites, comorbidities, and speculations on altered disease pathology for both infections, citing six references within this section. We would appreciate it if the reviewer allowed our text to remain as presented.

3.Consider rephrasing “... with about 80% of African population affected [13]” to clarify that a portion of the 80% represents asymptomatic individuals, thus reflecting previous exposure (sero-prevalence) to enhance clarity.

**Response:** We thank the reviewer for this comment. We have revised the text to indicate that *H. pylori* infections can be acquired during childhood and that prevalence may reach up to 80%. This revision reflects the fact that *H. pylori* typically persists chronically after acquisition, usually in early life, and that individuals with detectable antibodies are considered presumptively infected or may require clinical evaluation

4.The manuscript contains some important limitations which are not discussed in the manuscript. Kindly consider including a limitation section to comprehensively discuss this.

Reviewer: we thank the reviewer for this comment, we have now included a limitation section

Minor comments
1.It appears that some of the previous comments, specifically minor comments 1, 22 & 24, were not implemented in the revised manuscript. Kindly consider giving due consideration to these important points.

**Response:** We thank the reviewer for this comment. Previous comment 22 referred to the use of prevalence ratios (PR) versus odds ratios (OR). We apologize for missing this in the earlier review. Our primary objective was to describe co-infection patterns, which we addressed using basic descriptive statistics. The secondary objective was to examine whether infection patterns were associated with age or gender. In this context, ORs obtained via logistic regression provide a valid measure of association. In contrast, prevalence ratios or risk ratios compare the proportion of individuals with an outcome between groups, providing a measure of relative prevalence rather than the strength of association.

Regarding comment 24, we have provided the dataset as a supplementary file and included the analysis code. While we are not obliged to provide the scripts, readers interested in obtaining them can make a reasonable request to the corresponding author. Lastly, we would like to retain the list of keywords used

2.Please verify that the correct supplementary table has been uploaded, as the current version appears to contain missing participant data from line 301 onward. Additionally, the lack of standardized variable names and the absence of a codebook describing these variables make the dataset somewhat difficult to interpret. It is also worth reiterating that the R scripts used for the analysis have not been deposited in an open-access repository, which would aid a more comprehensive review and promote reproducible science for researchers interested in conducting similar studies in the future.

Response: we have provided the dataset as a supplementary file and included the analysis code. While we are not obliged to provide the scripts, readers interested in obtaining them can make a reasonable request to the corresponding author.

3. Although the acronym “WASH” was introduced in the abstract, it would be appropriate to reintroduce it at the first mention in the main text before subsequent use.

Response: we thank the reviewer for this comment. We have revised it, please see line 105

4.Please italicize Schistosoma at line 54, and ensure that all generic and specific names are consistently italicized throughout the manuscript for scientific accuracy.

Response: we thank the reviewer for this comment. We have revised it,

5.The use of “GC” in line 69 is unnecessary, as the term seems to appear only once throughout the manuscript.

Response: we thank the reviewer for this comment. We have revised it,

6.The use of “N” and “%” after each variable is unnecessary in the Tables, since a column is already dedicated to “N”. Instead, I guess you may include a footnote stating, for example, “values correspond to N (%)”. The same approach could be applied for Tables 2 and 3)

Response: we thank the reviewer for this comment. We have revised it,
